# Technology evaluations are associated with psychological need satisfaction across different spheres of experience: an application of the METUX scales

**DOI:** 10.3389/fpsyg.2023.1092288

**Published:** 2023-05-18

**Authors:** Ryan Burnell, Dorian Peters, Richard M. Ryan, Rafael A. Calvo

**Affiliations:** ^1^Leverhulme Centre for the Future of Intelligence, University of Cambridge, Cambridge, United Kingdom; ^2^Dyson School of Design Engineering, Imperial College London, London, United Kingdom; ^3^Institute for Positive Psychology and Education, Australian Catholic University, Sydney, NSW, Australia; ^4^College of Education, Ewha Womans University, Seoul, Republic of Korea

**Keywords:** Self-Determination Theory, human computer interaction (HCI), motivation, wellbeing, technology design

## Abstract

**Introduction:**

Digital technologies have the capacity to impact psychological wellbeing in both positive and negative ways. Improving technologies with respect to wellbeing requires nuanced understanding of this impact and reliable ways to measure it. Here, we aim to further this understanding by investigating the relations between psychological needs and people's evaluations of technologies (with respect to satisfaction, usability, and measures of value).

**Method:**

Across two studies with 1,521 participants, we improved and validated four scales that were first put forward as part of the METUX model of technology interaction. These scales measure psychological needs in the life, behavior, task, and interface spheres of experience. We applied these scales to four separate technologies (Facebook, TikTok, Blackboard, and Moodle), and examined the relationships between people's need satisfaction and frustration in the four spheres of experience and their overall evaluations of the technologies.

**Results and discussion:**

Each of the four scales had good psychometric properties across the four technologies. For each sphere of experience, psychological need satisfaction and frustration were associated with standard measures of usability and user satisfaction, and correlation patterns supported the METUX model and its approach to differentiating spheres of technology experience.

## 1. Introduction

Digital technologies are playing an increasingly important role in all aspects of modern life, from how we learn, work, and consume the news, to how we interact with others. Technologies therefore have tremendous potential to help improve people's lives. For example, learning management systems such as Moodle and Blackboard have been shown to increase student engagement and improve learning outcomes (Poondej and Lerdpornkulrat, 2019; Ismail et al., 2020). Devices such as Fitbits can help motivate people to exercise more regularly (Nuss et al., 2021), and video calling technologies such as Facetime have helped people maintain connections with others during the disruptions of the COVID-19 pandemic. However, technologies also have the potential to cause considerable harm. As one recent example, leaked internal research suggests that Instagram often has a negative impact on the self-esteem and mental health of young people who use it (Wells et al., 2021).

The effects of any given technology on people's motivation and wellbeing are likely to depend on a number of factors, including the extent to which that technology satisfies psychological needs. According to Self-Determination Theory (Deci and Ryan, 2000), people have three basic psychological needs that mediate the effects of social contexts, including technology, on wellbeing and motivation: autonomy, the feeling of having volition and choice over one's decisions and behaviors; competence, the feeling of being capable and effective at the activities one engages in; and relatedness, the feeling of being connected to other people (Deci and Ryan, 2000). The satisfaction of these three needs is associated with sustained engagement and wellbeing, while frustration of these needs is associated with disengagement and ill-being (Vansteenkiste and Ryan, 2013). The effects of need satisfaction on motivation and engagement have been demonstrated in a variety of domains, including the workplace (Niemiec and Ryan, 2009), education (Cox and Williams, 2008), and healthcare (Gorin et al., 2014). More recently, this theory has been applied to the domain of technology to help understand the factors that affect people's motivation and engagement with technology. For example, studies have found that need satisfaction predicts engagement with digital learning platforms (Chiu, 2021) and social media apps (Lin, 2016; Gao et al., 2021), as well as enjoyment of and time spent playing video games (Ryan et al., 2006). These findings demonstrate how important it is for designers, researchers, and policymakers to consider the effects a technology might have on psychological needs as they develop or evaluate it.

There are reasons to think these effects are likely to be complex and multifaceted. The model for Motivation, Engagement and Thriving in User Experience (METUX; Peters et al., 2018) suggests that technologies can affect psychological needs across the different spheres of experience. For example, a social media app might satisfy the psychological need for autonomy in the sphere of the interface by providing users with various options for viewing content. The app might also make it easy to accomplish certain tasks, such as posting images or creating videos to share with friends, which might fulfill people's psychological need for competence. More broadly, the app might make it easier to engage in important behaviors, such as staying in touch with friends and family, thus boosting both competence and relatedness in the sphere of that behavior. However, if they compulsively overuse the app to the detriment of other important activities such as work or family time then the app might ultimately frustrate people's sense of autonomy over how they spend their time and hurt their relatedness in their life as a whole. As this example illustrates, a technology that satisfies needs in one sphere does not necessarily satisfy needs in other spheres.

For this reason, simply measuring psychological need satisfaction or frustration in one sphere—for example, by asking people in the moment about how competent they feel while using the app—is not sufficient to fully understand how a technology is affecting people's psychological needs. Instead, the complex and sometimes contradictory effects of technologies on psychological needs require that we understand need satisfaction and frustration at all these spheres of experience. To address this gap, Peters et al. introduced an initial set of four scales known as the Technology-based Experience of Need Satisfaction scales (TENS). Each of the TENS scales measures need satisfaction and frustration caused by a specific technology in a particular sphere of experience that the researchers identified as being potentially important for need satisfaction and for people's engagement with a technology—one scale measures satisfaction and frustration in relation to the technology interface itself, one in relation to performing a specific task with the technology, one in relation to a broad behavior the technology helps with, and one in relation to life in general. The researchers argued that technologies might have differing effects on need satisfaction across these four spheres, although they do not discount the possibility that other spheres might also be important. These scales provide a more nuanced way of evaluating the extent to which a technology might be satisfying or frustrating psychological needs. The potential value of measures such as these to designers and researchers is clear from a range of studies that have applied these scales across a range of domains, including the design of accessibility software for blind individuals, online volunteer programs, language learning systems, and conversational agents (Naqshbandi et al., 2020; Nurhas et al., 2020; Rudinger, 2020; Yang and Aurisicchio, 2021).

There remains, however, a lack of empirical data on how psychological need satisfaction and frustration at these different spheres of user experience are related to common technology outcome measures such as user satisfaction, and time spent using the technology. These measures are seen by industry as essential to drive product success and inform its development. If, as the METUX model suggests, the different spheres are all important, we should expect that need satisfaction and frustration for each sphere are related to how positively people evaluate a technology and how much they use it. Evidence of these relationships would (a) provide support for the METUX model and suggest that the effects of technologies on psychological needs are multi-faceted, and (b) demonstrate further value in measuring need satisfaction across different spheres of the user experience to inform the development of technologies.

Therefore, we sought to examine the relations between technology-based need satisfaction and user satisfaction. To do so, we first need robust scales that measure need satisfaction and frustration at each of the spheres of experience. The scales proposed in Peters et al. (2018) served as a good initial step toward this goal, but have several limitations. First, those initial scales did not separate need satisfaction and need frustration, which have been shown to have different effects on people's wellbeing and ill-being outcomes (e.g., Chen et al., 2015; Ryan and Deci, 2017; Warburton et al., 2020). Second, the psychometric properties of these scales remain unclear. The researchers demonstrated that the scales had acceptable internal consistency, but due to the limitations of sample size they did not evaluate the factor structure of the scales or test the predictive and convergent validity of the scales.

To address these limitations, we modified the scales proposed by Peters et al. (2018) and then validated these newer versions of the scales. Using those validated scales, we then examined effects of need satisfaction and frustration on user satisfaction for two different categories of technologies. The first category—social media apps—was selected because these technologies are widely used and have the potential to both satisfy and frustrate psychological needs—particularly autonomy and relatedness. The second category we selected was digital learning platforms (such as Blackboard and Moodle), which are particularly likely to affect competence. In addition, whereas people choose to use social media apps, they are typically forced to use digital learning platforms by their institution—a factor which has been shown to have a bearing on need satisfaction and other outcomes within other contexts (Ryan and Deci, 2017). By using these two distinct categories of technologies, we were able to test the sensitivity of the scales to differences in effects on psychological needs.

## 2. Study 1

In Study 1, we modified four scales from Peters et al. (2018) known as the Technology-based Experience of Need Satisfaction scales (TENS)—the TENS-Life, the TENS-Behavior, the TENS-Task, and the TENS-Interface to produce a revised version for each. Then, we established their psychometric properties by asking participants to complete these scales in relation to two kinds of technology: a social media app and virtual learning platform.

### 2.1. Method

#### 2.1.1. Participants

We obtained data separately from two groups of participants: people who use Facebook, and University students who use virtual learning platforms. These studies received ethical approval from Imperial College London's Science, Engineering and Technology Research Ethics Committee. Both studies were pre-registered. The data and materials for both studies can be found on the Open Science Framework (https://osf.io/wn2m7). Practical constraints made it difficult to recruit participants who use the same learning platform, so we recruited university students online through Prolific (www.prolific.co/) and asked them about the learning platform they used the most (e.g., Blackboard, Moodle, Canvas). Following the recommendations of MacCallum et al. (1999) regarding sample size for factor analyses, we aimed to collect data from 300 participants (150 participants who use Facebook and 150 participants who use virtual learning platforms). In total, 355 participants completed the study (147 who use Facebook and a further 210 who use virtual learning platforms). We excluded 2 participants who failed both attention checks, leaving us with a final sample of 353, of whom 97 were men, 253 were women, and five were non-binary. The mean age of the sample was 26.64 (*SD* = 9.41). It was also a well-educated sample, three participants reported their highest level of education as “some secondary school,” 163 as “finished secondary school,” 131 as “finished an undergraduate degree,” and 57 as “finished a postgraduate degree.” One person chose not to answer.

#### 2.1.2. Materials

Drawing on the scales from Peters et al. (2018) as well as from established measures of psychological needs such as the BPNSF (Chen et al., 2015), we created four scales to measure both psychological need satisfaction and frustration for each of the different spheres of experience. The Life, Behavior, and Task scales each consist of six subscales—one each measuring autonomy satisfaction, autonomy frustration, competence satisfaction, competence frustration, relatedness satisfaction, and relatedness frustration. The Interface scale consists of the same subscales excluding relatedness satisfaction and frustration—we reasoned that it would not make sense for the interface of a technology (i.e., the buttons, controls and navigation) to satisfy or frustrate people's sense of connection to others.^1^ All scale items are rated on a 7-point Likert scale from 1 (*strongly disagree*) to 7 (*strongly agree*). Each subscale produces an overall score calculated by taking the mean of all the items within that subscale, meaning that each of the TENS scales has six overall subscale scores, each ranging between 1 and 7. It is important to note that the frustration items are not reverse coded, so higher scores indicate greater frustration with the technology.

Sample items for each scale are displayed in Table 1 to demonstrate the differences between the spheres. As the table shows, researchers should substitute into each item the name of the technology, behavior, and tasks being investigated. Below is a summary of each scale—the full scales can be found in the Supplementary material.

**Table 1:** Example items from each scale demonstrating the differences between the spheres.

**Scale**	**Example autonomy satisfaction item**	**Example autonomy frustration item**
TENS-life	[Facebook] gives me more freedom to do what really interests me	[Facebook] makes it harder to find time to pursue what matters to me
TENS-behavior	[Facebook] provides me with different options for [keeping up with friends and family]	[Facebook] does not provide me with enough choice over how I [keep up with friends and family]
TENS-task	[Facebook] provides me with different options for [posting to my timeline]	[Facebook] does not provide me with enough choice over how I [post to my timeline]
TENS-interface	I can customize [Facebook] to suit my needs	[Facebook] does not let me use it in the ways I want to

Information in brackets depends on the technology being investigated and should be substituted as needed.

##### 2.1.2.1. TENS-Life Scale

This scale consisted of 38 items designed to measure the broad effects of a technology on the satisfaction and frustration of psychological needs in everyday life.

##### 2.1.2.2. TENS-Behavior Scale

This scale consisted of 31 items designed to measure the effects of a technology on the satisfaction and frustration of psychological needs relating to a particular behavior (e.g., keeping in touch with friends).

##### 2.1.2.3. TENS-Task Scale

This scale consisted of 31 items designed to measure the effects of a technology on the satisfaction and frustration of psychological needs relating to a particular technology-supported task (e.g., sharing a photo on Facebook).

##### 2.1.2.4. TENS-Interface Scale

This scale consisted of 16 items designed to measure the effects of a technology on the satisfaction and frustration of psychological needs when interacting with the interface of that technology (e.g., controls, buttons, and navigation).

#### 2.1.3. Procedure

For participants recruited on the basis of using Facebook, we first asked them how they use it (through the app and/or through a web browser). For participants who reported using the app, we told them that for the remainder of the study we were interested in their experiences of using the Facebook app. For participants who reported using Facebook only through the web browser, we told them we were interested in their experiences using Facebook through the web browser.

For participants recruited because they use virtual learning platforms, we first asked them which learning platform they had used the most (Blackboard, Moodle, Canvas, or other). We told participants that for the remainder of the study we were interested in their experiences using that most-used platform.

Next, we asked participants how often they use the relevant technology (Facebook or Blackboard/Moodle/Canvas; note that no participant selected “other”), how satisfied they are with it, and how likely they would be to recommend it to a friend or colleague. We then asked participants whether and how often they use the technology to complete two tasks—for Facebook the tasks were sending messages and sharing photos with others, and for the learning platforms the tasks were submitting assignments and finding and viewing course materials.

Then, participants completed each of the METUX scales (Life, Behavior, Task, Interface) in a randomized order. For the behavior scale, we asked Facebook participants about the behavior of keeping up with friends and family, and for learning platform participants we asked them about keeping up with your courses. For the Task scale, participants completed the scale twice, once for the each of the two tasks we had earlier asked them about. If participants reported they never use the technology to complete one of the tasks, they did not complete the Task scale for that task. Participants always completed the two task scales consecutively, in a random order. In addition, the life scale and one of the task scales included an attention check asking participants to select a specific point on the scale. Finally, we collected basic demographics—age, gender, and level of education.

### 2.2. Results

Of the 146 participants whom we asked about Facebook, 70 reported using only the app to access it, while a further 66 reported using both the app and website, and 10 reported using only the website. Of the participants we asked about learning platforms, 104 used Blackboard, 75 used Moodle, and 30 used Canvas.

Next, we examined the internal consistency of the subscales. We found that the Cronbach's alphas were excellent across the board (>0.75), with only a couple of exceptions—the autonomy frustration subscales in the behavior and task spheres, which showed only acceptable internal consistency (0.66 and 0.69, respectively; see the Supplementary material for a detailed breakdown).

Having established the subscales had good internal consistency, we next examined the factor structure of the scales. For each of the Life, Behavior, and Task scales, we conducted an exploratory factor analysis assuming six factors and using an oblique rotation (allowing factors to be correlated, as the literature clearly demonstrates that the three psychological needs are highly correlated with one another; e.g., Chen et al., 2015). For the interface scale, we conducted the same analysis except assuming only 4 factors because relatedness was not included in this scale. For the sake of brevity, we report the full results of the factor analyses in Supplementary material. Across all four scales, items loaded as expected, with one exception: for the task of sharing photos on Facebook some of the competence satisfaction and competence frustration loaded together (with opposite valence) on the same factor. This pattern could be interpreted as evidence that the Task scale does not adequately separate the two constructs. However, the items for these two factors loaded as expected on all three other tasks, which shows the data generally fit with the expected factor structure. Taken together, the data from this study suggest that the adapted scales have good reliability.

## 3. Study 2

Study 1 provided initial evidence that the scales have reasonable psychometric properties. In Study 2 we sought to replicate these findings in a larger sample to demonstrate the scales are robust. This study was pre-registered on the Open Science Framework (https://osf.io/wn2m7). We also aimed to make the scales more manageable by shortening each subscale based on the psychometric evidence. Then, we turned to our main research question: how do need satisfaction and frustration across different spheres of experience relate to people's evaluations of technologies? To address this question, we used a larger sample and a broader sampling of technologies within our two categories of social media apps and virtual learning platforms.

### 3.1. Method

#### 3.1.1. Participants

We wanted to ensure we had a sufficient sample for robust factor analysis for each technology, and that we could estimate the relationships between psychological needs and technology use with high precision. We therefore followed the recommendations of MacCallum et al. (1999) and Schönbrodt and Perugini (2013) and aimed to collect data from ~300 participants using each technology. In total, 307 who use Facebook, 307 who use TikTok, 321 who use Blackboard, and 305 who use Moodle completed the study online. We excluded 12 participants who failed both attention checks, leaving us with a final sample of 1,221 (378 men, 826 women, 13 non-binary, and 4 choosing not to answer). The mean age was 27.95 (SD = 9.26). In total, 19 participants reported their highest level of education as “some secondary school,” 589 as “finished secondary school,” 425 as “finished an undergraduate degree,” 172 as “finished a postgraduate degree,” and 16 chose not to answer.

#### 3.1.2. Procedure

The materials and procedure for Study 2 was the same as for Study 1, with the following exceptions:

We included an additional technology (TikTok) in order to have data from two different social media platforms. Therefore, we separately recruited four groups of participants: a group who uses Facebook, a group who uses TikTok, a group who uses Blackboard, and a group who uses Moodle. All of the items participants completed referred to the relevant technology.

In this study, we again asked participants about two tasks, one “active” and one “passive,” a differentiation that has shown to have an impact on wellbeing (Burke and Kraut, 2016). For Facebook, these tasks were browsing the news feed and posting on my timeline. For TikTok, the tasks were watching videos and posting videos. For Blackboard and Moodle, the tasks were submitting assignments and accessing course materials. For each task, participants first reported whether they use the technology to do that task. If yes, they then rated how often they do that task, how much they enjoy doing the task, the extent to which the task is time well spent, and the extent to which the technology helps them enjoy doing the task.

Participants then rated a series of items evaluating their experience of engaging in the relevant behavior. First, they rated how often they engage in the behavior, how easy it is for them to do, how much choice they feel they have over how they do it, how much they enjoy it, the extent to which the behavior is time well spent, and the extent to which the technology helps them enjoy doing the behavior.

We also added an array of questions to assess people's evaluations of the technology and how much time they spend using it. More specifically, at the beginning of the study, participants completed a series of items measuring their satisfaction with the technology. First, they rated the technology from 1 to 5 stars (based on the standard app store rating system). Then, they rated how satisfied they are with the technology and how likely they are to recommend it to others using the standard Net Promoter Score (Reichheld, 2003). To investigate the convergent validity of the Interface scale, participants completed the System Usability Scale—a well-established scale that measures the usability of a technology's interface (Brooke, 1996).

Finally, participants responded to a series of questions about the extent to which they consider the technology worthwhile, and their time using it as time well spent. Specifically, they rated the extent to which the technology makes their life better, how much it helps them enjoy life, how useful it is, how often they use it, and the hours per week they spend using it.

After completing these items, participants completed the TENS Life, Behavior, Task, and Interface scales as in Study 1.

### 3.2. Results

#### 3.2.1. Psychometric properties of the scales

First, we examined the internal consistency of the subscales. Internal consistency was excellent as shown by Cronbach's alphas, which were all above 0.75 (see the Supplementary material for a full breakdown). Next, we examined the factor structure of each scale using confirmatory factor analyses, with the parameters estimated using maximum likelihood. We evaluated the fit of the models using Root Mean Square Error of Approximation (RMSEA), Standardized Root Mean Square Residual (SRMR), Comparative Fit Index (CFI), and Tucker-Lewis index (TLI). See the Supplementary material for path diagrams. As the left-hand side of Table 2 shows, the models all had relatively good fit.

**Table 2:** Model fit of confirmatory factor analyses from Study 2.

**Scale**	**Full scales**				**Shortened scales**			
	**RMSEA**	**SRMR**	**CFI**	**TLI**	**RMSEA**	**SRMR**	**CFI**	**TLI**
TENS-life	0.079	0.057	0.87	0.86	0.067	0.056	0.96	0.94
TENS-behavior	0.062	0.052	0.93	0.93	0.044	0.030	0.98	0.98
TENS-task	0.079	0.083	0.90	0.89	0.063	0.051	0.97	0.96
TENS-interface	0.109	0.083	0.91	0.884	0.104	0.063	0.94	0.92

Each row displays the factor analysis for one scale. RMSEA, Root mean squared error; SRMR, Standardized Root Mean Square Residual (higher scores correspond to worse fit). CFI, comparative fit index; TLI, Tucker-Lewis index (higher scores correspond to better fit, with 1 being perfect fit). Task values were calculated by taking the mean values across both tasks for each technology.

#### 3.2.2. Shortened scales

In accordance with our pre-registered plan, we created a shorter version of each scale on the basis of the confirmatory factor analyses. We did so for two main reasons: first, to remove items that did not fit well psychometrically with the other items in that subscale; and second, to create a shorter, more manageable set of scales that designers, researchers, and policy makers would find more practical. Inspection of the reliability analyses revealed that shortening each subscale to three items would improve internal consistency while still capturing most of the variance explained by the full scales. We therefore selected three items from each subscale for the final, shortened versions, which can be found in the Supplementary material.

After creating these shortened versions of the scales, we examined their psychometric properties. As the right-hand side of Table 2 shows, these scales had excellent internal consistency. Moreover, when we conducted confirmatory factor analyses using the same approach as we had used with the full versions of the scales, the models again demonstrated a good fit (see the Supplementary material). The RMSEA for the interface scale was somewhat high (although the CFI, TLI, and SRMR were still acceptable), suggesting there might be some room to improve the reliability of this scale.

#### 3.2.3. Overall levels of need satisfaction and frustration across technologies

It is also useful to consider differences between the technologies on need satisfaction and frustration across the different spheres. Because the technologies evaluated are all relatively mature, well-resourced, and have been very widely adopted, we might expect them all to have relatively good interfaces and to be relatively useful for completing relevant tasks and behaviors.

**Figure 1:**
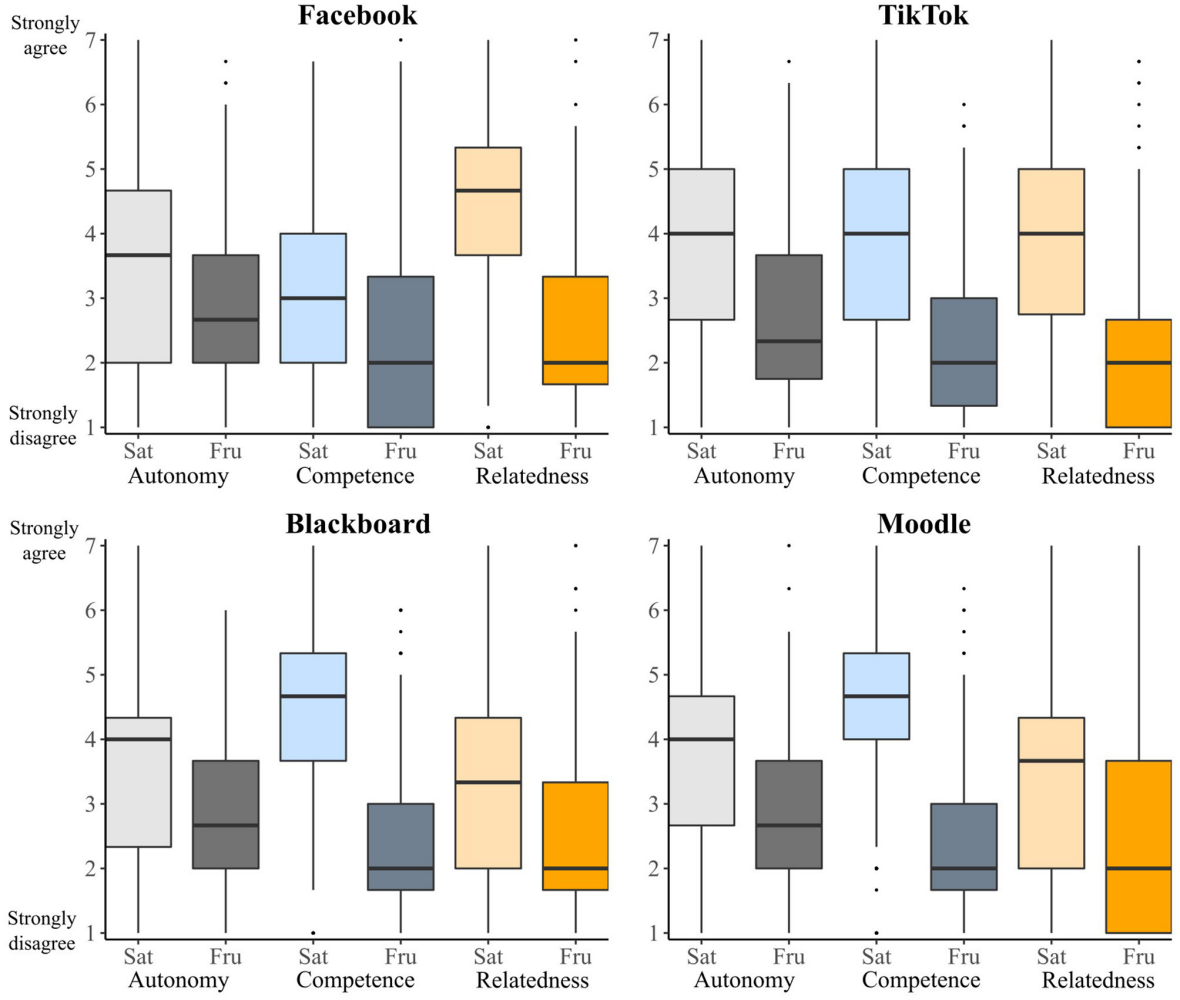
Box plots of psychological need ratings in the life sphere, split by technology. Autonomy ratings are displayed in gray, competence ratings are displayed in blue, and competence ratings are displayed in orange. Light-colored bars display satisfaction ratings, dark colored bars display frustration ratings.

Yet we can make clear predictions about how these different technologies might affect psychological needs in the life sphere. Given that Facebook and TikTok are designed to promote social interactions, we should expect these apps to satisfy relatedness more than Blackboard and Moodle. Indeed, as Figure 1 shows, we found that Facebook and TikTok were rated substantially higher on relatedness satisfaction than both Blackboard and Moodle. In contrast to our prediction for relatedness, we expected Blackboard and Moodle to satisfy people's sense of competence more than Facebook or TikTok because of the role these virtual learning platforms play in helping people complete their university courses. As expected, both Blackboard and Moodle were rated higher on competence satisfaction than Facebook and TikTok. Together, these findings demonstrate that different kinds of technologies tend to affect psychological needs in different ways.

We can also use these scales to examine differences between different technologies of the same kind. For example, Facebook was rated higher on relatedness satisfaction than TikTok—perhaps because it places greater focus on sharing and viewing the activities of friends and family. By contrast, TikTok was rated higher on competence satisfaction than Facebook. Further research scrutinizing these differences could help to illuminate the specific features of TikTok and Facebook that promote broad life-sphere competence and relatedness satisfaction.

**Figure 2:**
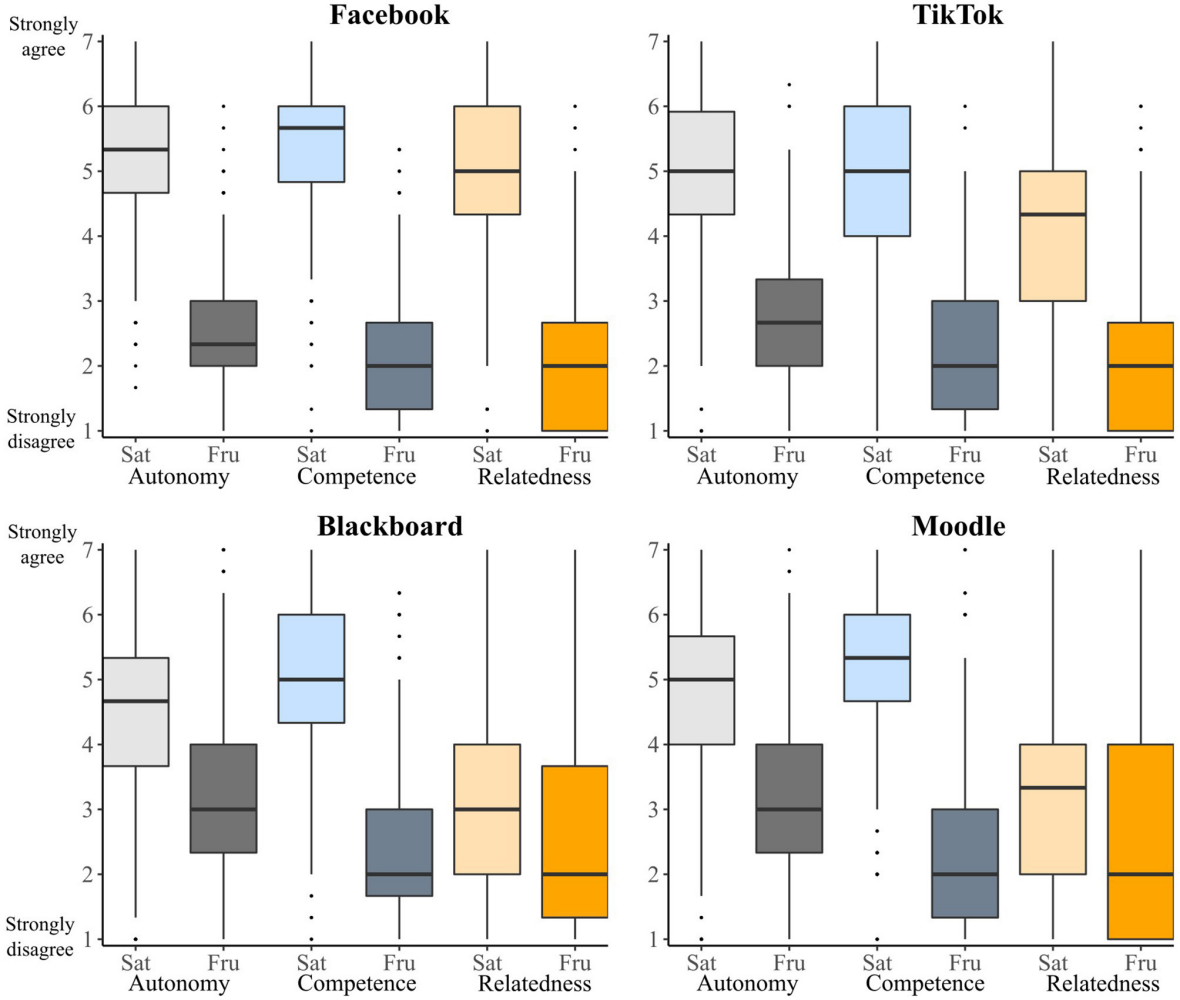
Box plots of psychological need ratings in the behavior sphere, split by technology. Autonomy ratings are displayed in gray, competence ratings are displayed in blue, and competence ratings are displayed in orange. Light-colored bars display satisfaction ratings, dark colored bars display frustration ratings.

One important prediction of the METUX model is that a technology can satisfy psychological needs in one sphere (e.g., in the sphere of the interface), but at the same time failing to satisfy that need in other spheres. We tested this prediction in several ways. We did so first by examining need satisfaction patterns in the behavior sphere and comparing those to the life sphere patterns described above. As Figure 2 shows, the effects of the four technologies on psychological needs in the behavior sphere differ in a number of ways to their effects on psychological needs in the life sphere. Most notably, all four technologies satisfy competence and autonomy far more in the behavior sphere (displayed in the top half of Figure 2) than they do in the life sphere (displayed in the top half of Figure 1). These findings fit with the hypothesis that technologies can affect psychological needs differently across different spheres of experience.

Stronger evidence for this hypothesis, though, would be the presence of participants who report high satisfaction of a psychological need in one sphere, but high frustration of that need in another sphere. Therefore, we examined (in an exploratory manner) the proportion of participants who reported high autonomy satisfaction in the interface sphere (above the midpoint), but also high autonomy frustration in the life sphere (above the midpoint). We chose this analysis because theoretical accounts suggest that engaging, well-designed technologies with easy-to-use interfaces can be paradoxically some of the most addictive and problematic technologies at a high level (Peters et al., 2018). The analyses revealed a substantial proportion of participants who fell into the subgroup reporting high need satisfaction in the interface sphere but high frustration in the life sphere-−9% of TikTok participants fell into this subgroup, along with 5% of Facebook participants, 5% of Blackboard participants, 3% of Moodle participants. We found a similar pattern with competence-−10% of Facebook participants reported high satisfaction in the interface sphere but high frustration at the life, along with 7% of TikTok participants, 3% of Blackboard participants and 4% of Moodle participants. Because the interface scale does not measure relatedness, we did not conduct an analysis for this need. These findings provide further support for the hypothesis that technologies can have conflicting effects on people's psychological needs across different spheres.

#### 3.2.4. Relations between need satisfaction/frustration and technology evaluations

Next, we examined the relations between people's evaluation of the technologies and their reports of need satisfaction and frustration as measured with the shortened versions of the TENS scales. Across all four spheres, we found that need satisfaction was associated with higher star ratings, higher satisfaction with the technology, and greater willingness to recommend the technology to others. By contrast, need frustration was associated with lower star ratings, lower satisfaction, and lower willingness to recommend the technology to others. On the whole, the relations between need frustration and technology evaluations tended to be weaker than between need satisfaction and technology evaluations (see Table 3 for a full breakdown of these correlations).

**Table 3:** Correlations from Study 2 between technology evaluations and need satisfaction and frustration ratings for each sphere.

**Subscale**	**Star rating**	**Satisfaction with technology**	**Willingness to recommend to others**
**Life**			
Autonomy satisfaction	0.40	0.42	0.46
Autonomy frustration	−0.19	−0.19	−0.14
Competence satisfaction	0.36	0.39	0.38
Competence frustration	−0.25	−0.26	−0.22
Relatedness satisfaction	0.30	0.32	0.37
Relatedness frustration	−0.23	−0.23	−0.23
**Behavior**			
Autonomy satisfaction	0.36	0.33	0.40
Autonomy frustration	−0.36	−0.34	−0.35
Competence satisfaction	0.35	0.38	0.37
Competence frustration	−0.27	−0.30	−0.31
Relatedness satisfaction	0.24	0.23	0.29
Relatedness frustration	−0.20	−0.22	−0.23
**Task**			
Autonomy satisfaction	0.33	0.31	0.37
Autonomy frustration	−0.38	−0.37	−0.37
Competence satisfaction	0.45	0.47	0.48
Competence frustration	−0.35	−0.35	−0.33
Relatedness satisfaction	0.31	0.28	0.37
Relatedness frustration	−0.22	−0.25	−0.26
**Interface**			
Autonomy satisfaction	0.45	0.44	0.46
Autonomy frustration	−0.38	−0.38	−0.37
Competence satisfaction	0.39	0.41	0.38
Competence frustration	−0.32	−0.32	−0.30

All correlations are significantly different from zero with *p* < 0.001.

Given these correlations, we next sought to more robustly evaluate how need satisfaction and frustration in each sphere are related to evaluations of the technology. To do so, we first created, for each sphere, one combined measure of need satisfaction by taking the mean of autonomy, competence, and relatedness satisfaction, and a corresponding combined measure of need frustration by taking the mean of autonomy, competence, and relatedness frustration. Using these combined measures, we conducted a linear regression for each technology, with need satisfaction and frustration for each sphere predicting overall satisfaction with the technology.

As Table 4 shows, the most relevant sphere of experience for predicting overall satisfaction with the technology varied across the technologies we investigated. More specifically, for both TikTok and Facebook, need satisfaction in the behavior sphere was the strongest predictor of predicted overall satisfaction, suggesting that the most important determinant of people's overall satisfaction with these technologies might be the apps' ability to help provide a sense of autonomy, competence, and relatedness through helping people keep up with friends and family. We also found interesting differences between the two social media technologies. For TikTok, but not Facebook, need frustration in the life sphere predicted overall satisfaction such that higher frustration was associated with lower overall satisfaction. By contrast, for Facebook, but not TikTok, need frustration in the task sphere predicted overall satisfaction such that higher frustration was associated with lower overall satisfaction. These findings could suggest that the harmful effects of TikTok on people's broad life needs are a greater concern than for Facebook, for which frustrations in performing specific tasks appear to be more central to overall satisfaction with the technology. One surprising finding was that need frustration in the behavior sphere predicted overall satisfaction across both TikTok and Facebook such that higher need frustration was associated with higher overall satisfaction. However, this finding could be an artifact of the data caused by the not insignificant negative correlations between the satisfaction and frustration scales. Indeed, a look at raw correlations between need frustration in the behavior sphere and overall satisfaction shows the expected negative relationships (*r* = −0.28 for TikTok, and *r* = −0.27 for Facebook).

**Table 4:** Beta weights from regressions for each technology with need satisfaction and frustration for each sphere predicting overall satisfaction with the technology.

**Predictor**	***TikTok***	**Facebook**	**Blackboard**	**Moodle**
**Life**				
Satisfaction	0.05	0.13	0.08	0.12
Frustration	−0.19^*^	−0.04	0.02	0.05
**Behavior**				
Satisfaction	0.30^***^	0.32^**^	−0.06	0.04
Frustration	0.23^*^	0.36^**^	−0.07	−0.09
**Task**				
Satisfaction	−0.11	−0.02	0.11	−0.02
Frustration	0.02	−0.26^*^	−0.16	−0.09
**Interface**				
Satisfaction	0.06	0.23	0.17^**^	0.30^**^
Frustration	−0.03	0.04	0.01	−0.03

^*^indicates *p* < 0.05, ^**^indicates *p* < 0.01, ^***^indicates *p* < 0.001.

The results for Blackboard and Moodle show quite a different picture. For these technologies, need satisfaction in the interface sphere was the strongest (and only significant) predictor of overall satisfaction with the technology. This finding suggests that people's opinions of learning management systems are primarily driven by their experiences with the interface.

Taken together, these findings show that the relative importance of the different spheres varies from technology to technology, and that the spheres are tapping into different aspects of need satisfaction and frustration. It's important to note, however, that the raw correlations suggest that all spheres are at least somewhat related to overall technology satisfaction across all four technologies. In turn, these findings show that granular measurements of psychological needs across the different spheres of experience are required for a full understanding of how a technology is affecting the satisfaction or frustration of people's psychological needs.

Next, we explored the relations between measures specific to each sphere and people's need satisfaction and frustration ratings within that sphere. First, we examined the correlations between the Interface subscales and participants' ratings of usability on the System Usability Scale—to the extent that usability is related to feelings of competence with respect to an interface, expecting the usability score to be positively related to competence satisfaction and negatively related to competence frustration. In line with these expectations, we found a strong positive correlation between competence satisfaction in the interface sphere and the usability score, *r*_(1, 221)_ = 0.76, *p* < 0.001. We also found a strong negative correlation between competence frustration in the interface sphere and usability, *r*_(1, 221)_ = −0.73, *p* < 0.001. As expected, the correlations between the usability score and competence satisfaction at other spheres of experience were substantially weaker (*r*s < 0.6). These correlations demonstrate convergent validity with a widely used usability measure.

Next, we examined the relations between the subscales of the TENS Task Scale and participants' reports of their experience engaging in behaviors using their respective technology. We found small-to-moderate correlations between people's need ratings and their reports of how easy the task is, how much they enjoy the task, and the extent to which doing the task is time well spent. The correlations between need satisfaction and how often people do the task were small, at best (see Supplementary material for a full breakdown of these results).

Next, we examined the relations between the subscales of the TENS Behavior Scale and participants' reports of their experience engaging in the relevant behavior. We found small-to-moderate correlations between people's need ratings and their reports of how easy the behavior is, how much choice they have over the behavior, and how much they enjoy the behavior. We did not find any significant associations between people's need ratings and their reports of how often they do the behavior, nor with their agreement that the behavior is time well spent (see Supplementary material).

Finally, we examined the relations between people's need ratings in the life sphere and their general ratings about how the technology affects their life. We found that need satisfaction was positively correlated with the belief that the technology makes their life better, helps them enjoy life, and is useful to them. By contrast, need frustration was negatively correlated with those same variables (see the Supplementary material for a full breakdown). However, correlations between need satisfaction and frustration and how often people used the technology were small, at best. Therefore, it seems likely that frequency of use is divergent from these positive and negative experiences, and likely driven by other factors.

## 4. General discussion

Across two studies and 1,521 participants, we investigated the relations between basic psychological needs and people's evaluations of technologies. To do so, we created four scales to measure psychological need satisfaction and frustration in the life, behavior, task, and interface spheres of technology use. We found that each of the scales had good psychometric properties when applied to four separate technologies (Facebook, TikTok, Blackboard, and Moodle). Across each of the four spheres, need satisfaction and frustration were correlated with people's satisfaction with the technologies.

Our findings build on and extend work examining the effect of psychological needs on motivation and engagement with different technologies (e.g., Ryan et al., 2006; Lin, 2016; Chiu, 2021; Gao et al., 2021). More, specifically, our findings provide empirical support for the METUX model of technology interactions and highlight the importance of measuring psychological needs across different spheres of experience (Peters et al., 2018). We found that need satisfaction and frustration in all four spheres were related to user evaluations of Facebook, TikTok, Blackboard, and Moodle. For example, our data suggest that people's satisfaction with Facebook relates to both how much autonomy it gives them over how they post to the news feed, and how much autonomy it gives them over how they spend their time more broadly. Moreover, regression analyses showed that the sphere that most strongly predicted overall satisfaction with the technology differed across the four technologies. In addition, we found that a sizeable minority of participants reported high need satisfaction in the interface sphere, but high need frustration in the life sphere. These findings suggest that the scales are tapping into different aspects of need satisfaction that are important for people's motivation and engagement with different technologies.

Of particular note is the finding that people's evaluations of the technologies were related to the extent to which they reported that the technology supported their psychological needs in the life and behavior spheres. This finding suggests that people's evaluations of a technology depend on more than just their moment-to-moment user experience (Marangunić and Granić, 2015)—instead, these evaluations also depend on the broader effects the technologies are having on people's lives. In the context of apps like Facebook and TikTok, this finding suggests that a focus on need satisfaction in the life sphere—for example, by ensuring the apps encourage positive interactions that increase feelings of relatedness (Lin, 2016)—could boost people's satisfaction with those technologies and increase their willingness to recommend the technologies to others. More generally, this finding suggests that designing for motivation and wellbeing can have tangible benefits both for users and for the companies who design the technologies.

It is worth noting, however, that only a small number of effects were significant for each technology and that the regression coefficients were relatively small. For this reason, one might be tempted to conclude that need satisfaction has only small effects on people's overall technology evaluations. Naturally, we can expect that in most cases the effects of any one technology on people's overall wellbeing are likely to be small, but there are still good examples where they might be meaningful (e.g., in the case of social media apps). Part of our motivation for creating these scales is that much of the assessment done in technology design is focused on people's immediate interface or task experiences, neglecting the broader behavior and life level experiences. However, it's important to remember that these coefficients convey the effects of need satisfaction in a particular sphere after controlling for need frustration in that same sphere as well as need satisfaction and frustration in all other spheres. Given the similarities between the measures and the not insignificant collinearity between them, the fact that we still found significant effects demonstrates the important effects of experiences across the different spheres. Moreover, an examination of the raw correlations reveals quite strong relationships between need satisfaction (and frustration) and overall technology evaluations. Of course, further work is needed to establish causality in these relationships, but the correlations suggest that need satisfaction is quite tightly coupled with people's overall evaluations of technologies.

In these studies, we were not able to directly measure people's behavioral choices regarding technology usage, nor the effects of the technologies on people's wellbeing. It is therefore important that future work examines the relationship between need satisfaction across the different spheres of experience and people's wellbeing and behaviors to elucidate the downstream consequences of these technology experiences. We might also expect the different spheres to affect people's behaviors in different ways, in a similar way to how attitudes have different effects on behavior depending on their specificity (Ajzen and Fishbein, 1977). For example, life-level satisfaction might have the strongest effects on broad choices about whether to adopt or stop using a technology, whereas interface-level satisfaction might have greater effects on how and how frequently people use the technology.

These findings also have broader theoretical implications for how we understand psychological needs. In particular, the findings raise the possibility that psychological needs can be affected in multifaceted and conflicting ways across different spheres of experience. Here, we demonstrated this possibility in relation to technologies. But we might see similar patterns in other contexts, too. For example, people might feel that their job gives them autonomy over how they perform day-to-day work tasks, but that the job ultimately impairs their autonomy over their life choices. Likewise, people might feel a sense of relatedness while they interact with a friend or partner, but at the same time feel that the relationship frustrates their connectedness with other people. If so, it is important that studies investigating psychological needs carefully consider and specify the effects they are interested in measuring.

To assist this measurement effort in the context of technologies, we derived four new and briefer TENS scales that can measure the effects of technology on need satisfaction across different spheres of experience. These scales build on the initial scales proposed in Peters et al. (2018) in two key ways. First, these scales have been more thoroughly validated herein. Specifically, the scales had good measurement properties across two markedly different categories of technology, suggesting the scales could be used across a range of domains. The improvements reported here address concerns raised by researchers who have implemented the scales in the past—namely that some items do not fit neatly into the expected factor structure, and that the scales might be lacking in convergent validity in some cases (Jeno et al., 2021). Second, these new scales separately evaluate need satisfaction and frustration for each sphere of experience, which allows for more granularity in the assessment of how technologies affect psychological needs.

We hope that the scales might be useful for a variety of applications. First, these scales could be used by researchers to further our understanding of how technologies, in general, affect people's psychological needs and wellness. Second, the scales (and contextualized adaptations of them) could guide the design of new technologies by showing potential effects on people's psychological needs—as need frustration in any of the spheres would indicate potential harms to both engagement and wellbeing that the designers should address. Third, the scales could be used by designers who are changing the features of an existing technology. By comparing the impact of old and new designs on need satisfaction, designers could determine whether the proposed change is beneficial or harmful in terms of psychological need fulfillment, wellbeing, and by extension, user satisfaction. Finally, the scales could be used to compare different technologies within the same domain—for instance, our data show that Facebook tended to satisfy relatedness more than TikTok in the life sphere. This seems logical since TikTok is more narrowly focused on video content consumption, whereas Facebook offers more features for direct interaction with others. However, if designers of either platform wanted to expand their feature set to increase support for human connection (as Instagram arguably did when they added features such as messaging and stories) then they could use these measures to evaluate the success of those new features.

Based on the results of the confirmatory factor analyses, we selected three items for each subscale to ensure the scales are relatively short. But depending on the technology being investigated, different combinations of items might be useful to include. For example, if a technology has a component that involves assisting others—as in the case of collaboration tools such as Trello (for work collaboration) or Freecycle (for donating) —then items such as “Using this technology to [do the behavior] makes it easier to contribute or help other people” might be worth including. Including items such as this one should be feasible given that all items in the longer versions of the scales loaded reasonably strongly on the relevant factors. Moreover, depending on how the scales are being used, it might only be necessary to include some subscales. For example, in the case of a banking app or fitness band, which are unlikely to have major effects on relatedness, one might exclude the relatedness items for the sake of brevity.

Of course, the studies reported here have several limitations. First, because items need to be slightly modified to fit the technology of interest, the psychometric properties of the scales may change across different technologies. It is therefore important for researchers to check the psychometric properties of the scales when using them for a new technology. Second, the cross-sectional design means the directions of the associations between people's evaluations of technologies and their reports of need satisfaction are not clear. Although it would fit with theoretical models of motivation and engagement for need satisfaction to affect technology evaluation, it is also possible that people who generally have positive views of a technology tend to, in turn, rate that technology as supporting psychological needs. Third, we applied measurements to users who had already adopted these technologies. As a result, the impact of need satisfaction and frustration in the adoption and interface spheres are likely less salient in this current data, as these are users who have already adopted, and likely mastered the basic interfaces. Future studies looking at METUX measures across the life span of user experience, from adoption to long term use, may be especially in helpful predicting issues of attrition or churn.

It also remains unclear how need satisfaction and frustration relate to evaluations in other technology domains. For instance, none of our target technologies tended to frustrate psychological needs to a high degree—which is perhaps why these technologies are so widely used. It may be that need frustration plays a bigger role in technology evaluations than we saw in our data, particularly in early phases of exposure as consumers select and curate the technologies they use. Our data might also underestimate need frustration on the whole, because our samples consisted only of current users. Therefore, people who have abandoned a technology all together —for example, because it frustrates psychological needs—are not reflected in these samples.

Finally, a key limitation of the scales is that they rely on retrospective self-report. Future research using experience sampling and longitudinal designs will help to refine these models, and better pinpoint potential causal processes.

### 4.1. Conclusion

Taken together, the studies reported here provide evidence for the value in measuring need satisfaction across different spheres of experience, and our data suggest the modified TENS scales presented herein are a practical and flexible way of doing so. It is our hope that these measures will help designers, researchers, and policy makers to better understand the effects of technologies on psychological needs—an understanding that is central to ensuring that technologies foster, rather than hinder, wellbeing.

## Data availability statement

The datasets presented in this study can be found in online repositories. The names of the repository/repositories and accession number(s) can be found below: https://osf.io/wn2m7.

## Ethics statement

The studies involving human participants were reviewed and approved by Imperial College London's Science, Engineering and Technology Research Ethics Committee. The patients/participants provided their written informed consent to participate in this study.

## Author contributions

RB, DP, RR, and RC developed the study concept and design together. RB organized data collection and performed all data analyses. RB drafted the manuscript with RC. DP and RR provided critical revisions. All authors contributed to the article and approved the submitted version.

## Footnotes

^1^Even in the case of technologies such as conversational interfaces, it is not the interface itself supporting relatedness, but rather the interaction *task*, such as interacting with a virtual agent or chatting with a friend. There are some exceptions, such as for very specific visual features (e.g., emojis) or for experimental devices that provide tactile experiences designed for connection, but for the vast majority of current technologies, questions about how navigation and controls satisfy relatedness would seem incongruous.
