# Supplementary material for: Technology evaluations are associated with psychological need satisfaction across different spheres of experience: an application of the METUX scales
Source: Front Psychol. 2023 May 18;14:1092288. doi: 10.3389/fpsyg.2023.1092288 (PMC10232989; doi:10.3389/fpsyg.2023.1092288)

Supplemental Materials: Technology ratings are associated with psychological need satisfaction across different spheres of experience: An application of the METUX Scales

# Initial scales from Experiment 1

| TENS-Life Scale (Initial long version) |
| --- |
| **Autonomy** |
| *Support* |
| [Technology name] gives me more choice in the things I do |
| [Technology name] helps me use my time to pursue what matters to me |
| [Technology name] helps me make choices that express who I really am |
| [Technology name] gives me more freedom to do what really interests me |
| [Technology name] makes it easier for me to choose what I do with my life |
| *Frustration* |
| [Technology name] makes me feel pressured to do things I wouldn’t choose to do |
| I feel pressured to use [Technology name] |
| [Technology name] makes it harder to find time to pursue what matters to me |
| [Technology name] gets in the way of my ability to choose what I do with my life |
| I feel obliged to use [Technology name] more than I’d like to |
|  |
| **Competence** |
| *Support* |
| [Technology name] helps me feel confident in my abilities |
| [Technology name] helps me feel competent at things I do |
| [Technology name] helps me operate effectively in my life |
| [Technology name] helps me feel I can successfully complete difficult tasks |
| [Technology name] helps me feel capable of achieving my goals |
| [Technology name] helps me to learn and grow as a person |
| [Technology name] helps me improve my abilities |
| *Frustration* |
| [Technology name] makes me feel insecure about my abilities |
| [Technology name] makes me doubt whether I am a capable person |
| [Technology name] makes it difficult for me to overcome challenges |
| [Technology name] makes it difficult for me to achieve my goals |
| [Technology name] hinders my ability to learn and grow as a person |
| [Technology name] makes it difficult for me to improve my abilities |
| [Technology name] makes it difficult for me to operate effectively in my life |
|  |
| **Relatedness** |
| *Support* |
| [Technology name] helps me feel close to people who are important to me |
| [Technology name] helps me feel connected to other people in a meaningful way |
| [Technology name] helps me feel that other people care about me |
| [Technology name] helps me feel like I am part of a larger community |
| [Technology name] makes it easier for me to help other people |
| [Technology name] helps me feel like I have something to contribute |
| [Technology name] helps me feel supported by other people |
|  |
| *Frustration* |
| [Technology name] makes it harder to connect to people in a meaningful way |
| [Technology name] makes me feel like other people don’t care about me |
| [Technology name] makes me feel like I don’t matter much to other people |
| [Technology name] makes me feel excluded |
| [Technology name] makes me feel lonely |
| [Technology name] makes it harder for me to help other people |
| [Technology name] makes it harder for me to engage with my community |
| Note. All items rated from 1 (*Strongly disagree*) to 7 (*Strongly agree*) |

| TENS-Behaviour Scale (Initial long version from Experiment 1) |
| --- |
| **Autonomy** |
| *Support* |
| This technology provides me with different options for [doing the behaviour] |
| This technology gives me freedom to [do the behaviour] the way I want |
| This technology helps me have more choice over how I [do the behaviour] |
| This technology makes me want to [do the behaviour] |
| Using this technology to [do the behaviour] aligns with my values |
| *Frustration* |
| This technology doesn’t provide me with enough choice over how I [do the behaviour] |
| This technology forces me to [do the behaviour] in ways I don’t like |
| This technology restricts my options for [doing the behaviour] |
| This technology makes me feel pressure to [do the behaviour] |
| Using this technology to [do the behaviour] conflicts with my values |
|  |
| **Competence** |
| *Support* |
| This technology makes it easier to [do the behaviour] |
| This technology helps me feel confident in my ability to [do the behaviour] |
| This technology helps me [do the behaviour] effectively |
| This technology is useful for [doing the behaviour] |
| This technology helps me get better at [doing the behaviour] |
| *Frustration* |
| This technology makes it difficult to [do the behaviour] |
| This technology gets in the way of my ability to [do the behaviour] |
| ­­­I struggle to use this technology to [do the behaviour] |
| This technology makes me lose confidence in my ability to [do the behaviour] |
| This technology makes it harder for me to improve at [doing the behaviour] |
|  |
| **Relatedness** |
| *Support* |
| When I [do the behaviour] with this technology, it makes me feel close to people who are important to me |
| When I [do the behaviour] with this technology, it makes me feel connected to other people |
| When I [do the behaviour] with this technology, it makes me feel that people care about me |
| When I [do the behaviour] with this technology, it makes me feel like I am part of a larger community |
| Using this technology to [do the behaviour] makes it easier to contribute or help other people |
|  |
| *Frustration* |
| When I [do the behaviour] with this technology, it makes me feel disconnected from other people |
| When I [do the behaviour] with this technology, it makes me feel that people don’t care about me |
| When I [do the behaviour] with this technology, it makes me feel excluded or isolated |
| When I [do the behaviour] with this technology, it makes me feel lonely |
| When I [do the behaviour] with this technology, it makes me feel like I don’t matter to other people |
| Using this technology to [do the behaviour] makes it harder to contribute or help other people |
| When I [do the behaviour] with this technology, it makes me feel disconnected from other people |
| Note. All items rated from 1 (*Strongly disagree*) to 7 (*Strongly agree*) |

| TENS-Task Scale (Initial long version from Experiment 1) |
| --- |
| **Autonomy** |
| *Support* |
| This technology provides me with different options for [doing the task] |
| This technology gives me freedom to [do the task] the way I want |
| This technology helps me have more choice over how I [do the task] |
| This technology makes me want to [do the task] |
| Using this technology to [do the task] aligns with my values |
| *Frustration* |
| This technology doesn’t provide me with enough choice over how I [do the task] |
| This technology forces me to [do the task] in ways I don’t like |
| This technology restricts my options for [doing the task] |
| This technology makes me feel pressure to [do the task] |
| Using this technology to [do the task] conflicts with my values |
|  |
| **Competence** |
| *Support* |
| This technology makes it easier to [do the task] |
| This technology helps me feel confident in my ability to [do the task] |
| This technology helps me [do the task] effectively |
| This technology is useful for [doing the task] |
| This technology helps me get better at [doing the task] |
| *Frustration* |
| This technology makes it difficult to [do the task] |
| This technology gets in the way of my ability to [do the task] |
| ­­­I struggle to use this technology to [do the task] |
| This technology makes me lose confidence in my ability to [do the task] |
| This technology makes it harder for me to improve at [doing the task] |
|  |
| **Relatedness** |
| *Support* |
| When I [do the task] with this technology, it makes me feel close to people who are important to me |
| When I [do the task] with this technology, it makes me feel connected to other people |
| When I [do the task] with this technology, it makes me feel that people care about me |
| When I [do the task] with this technology, it makes me feel like I am part of a larger community |
| Using this technology to [do the task] makes it easier to contribute or help other people |
|  |
| *Frustration* |
| When I [do the task] with this technology, it makes me feel disconnected from other people |
| When I [do the task] with this technology, it makes me feel that people don’t care about me |
| When I [do the task] with this technology, it makes me feel excluded or isolated |
| When I [do the task] with this technology, it makes me feel lonely |
| When I [do the task] with this technology, it makes me feel like I don’t matter to other people |
| Using this technology to [do the task] makes it harder to contribute or help other people |
| When I [do the task] with this technology, it makes me feel disconnected from other people |
| Note. All items rated from 1 (*Strongly disagree*) to 7 (*Strongly agree*) |

| TENS-Interface Scale (Initial long version from Experiment 1) |
| --- |
| **Autonomy** |
| *Support* |
| This technology provides me with useful options and choices |
| This technology gives me freedom to use it the way I want |
| I can customise this technology to suit my needs |
| This technology’s interface is flexible |
| *Frustration* |
| This technology doesn’t provide me with enough options and choices |
| The technology doesn’t let me use it in the ways I want to |
| It’s difficult to customise the technology |
| This technology’s interface is restrictive or controlling |
|  |
| **Competence** |
| *Support* |
| I find it easy to use this technology |
| I feel confident in my ability to use this technology |
| I am capable of using this technology effectively |
| This technology’s interface is intuitive |
| *Frustration* |
| I find it hard to use this technology |
| I have doubts about my ability to use this technology |
| I struggle to use this technology effectively |
| This technology’s interface is confusing |
| Note. All items rated from 1 (*Strongly disagree*) to 7 (*Strongly agree*) |

# Final, Shortened scales

| Appendix 1. Final shortened TENS Life Scale |
| --- |
| **Autonomy** |
| *Support* |
| [Technology name] helps me make choices that express who I really am |
| [Technology name] gives me more freedom to do what really interests me |
| [Technology name] makes it easier for me to choose what I do with my life |
| *Frustration* |
| [Technology name] makes it harder to find time to pursue what matters to me |
| [Technology name] gets in the way of my ability to choose what I do with my life |
| I feel obliged to use [Technology name] more than I’d like to |
|  |
| **Competence** |
| *Support* |
| [Technology name] helps me feel I can successfully complete difficult tasks |
| [Technology name] helps me feel capable of achieving my goals |
| [Technology name] helps me improve my abilities |
| *Frustration* |
| [Technology name] makes it difficult for me to overcome challenges |
| [Technology name] makes it difficult for me to achieve my goals |
| [Technology name] makes it difficult for me to improve my abilities |
| **Relatedness** |
| *Support* |
| [Technology name] helps me feel connected to other people in a meaningful way |
| [Technology name] helps me feel that other people care about me |
| [Technology name] helps me feel like I have something to contribute |
|  |
| *Frustration* |
| [Technology name] makes me feel like other people don’t care about me |
| [Technology name] makes me feel like I don’t matter much to other people |
| [Technology name] makes me feel excluded |
| Note. All items rated from 1 (*Strongly disagree*) to 7 (*Strongly agree*) |

| Appendix 2. Final shortened TENS Behaviour Scale |
| --- |
| **Autonomy** |
| *Support* |
| This technology provides me with different options for [doing the behaviour] |
| This technology gives me freedom to [do the behaviour] the way I want |
| This technology helps me have more choice over how I [do the behaviour] |
| *Frustration* |
| This technology doesn’t provide me with enough choice over how I [do the behaviour] |
| This technology forces me to [do the behaviour] in ways I don’t like |
| This technology restricts my options for [doing the behaviour] |
|  |
| **Competence** |
| *Support* |
| This technology helps me feel confident in my ability to [do the behaviour] |
| This technology helps me [do the behaviour] effectively |
| This technology is useful for [doing the behaviour] |
| *Frustration* |
| This technology gets in the way of my ability to [do the behaviour] |
| ­­­I struggle to use this technology to [do the behaviour] |
| This technology makes me lose confidence in my ability to [do the behaviour] |
|  |
| **Relatedness** |
| *Support* |
| When I [do the behaviour] with this technology, it makes me feel close to people who are important to me |
| When I [do the behaviour] with this technology, it makes me feel connected to other people |
| When I [do the behaviour] with this technology, it makes me feel that people care about me |
|  |
| *Frustration* |
| When I [do the behaviour] with this technology, it makes me feel excluded or isolated |
| When I [do the behaviour] with this technology, it makes me feel lonely |
| When I [do the behaviour] with this technology, it makes me feel like I don’t matter to other people |
| Note. All items rated from 1 (*Strongly disagree*) to 7 (*Strongly agree*) |

| Appendix 3. Final shortened TENS Task Scale |
| --- |
| **Autonomy** |
| *Support* |
| This technology provides me with different options for [doing the task] |
| This technology gives me freedom to [do the task] the way I want |
| This technology helps me have more choice over how I [do the task] |
| *Frustration* |
| This technology doesn’t provide me with enough choice over how I [do the task] |
| This technology forces me to [do the task] in ways I don’t like |
| This technology restricts my options for [doing the task] |
|  |
| **Competence** |
| *Support* |
| This technology helps me feel confident in my ability to [do the task] |
| This technology helps me [do the task] effectively |
| This technology is useful for [doing the task] |
| *Frustration* |
| This technology gets in the way of my ability to [do the task] |
| ­­­I struggle to use this technology to [do the task] |
| This technology makes me lose confidence in my ability to [do the task] |
|  |
| **Relatedness** |
| *Support* |
| When I [do the task] with this technology, it makes me feel close to people who are important to me |
| When I [do the task] with this technology, it makes me feel connected to other people |
| When I [do the task] with this technology, it makes me feel that people care about me |
|  |
| *Frustration* |
| When I [do the task] with this technology, it makes me feel excluded or isolated |
| When I [do the task] with this technology, it makes me feel lonely |
| When I [do the task] with this technology, it makes me feel like I don’t matter to other people |
| Note. All items rated from 1 (*Strongly disagree*) to 7 (*Strongly agree*) |

| Appendix 4. Final shortened TENS Interface Scale |
| --- |
| **Autonomy** |
| *Support* |
| This technology gives me freedom to use it the way I want |
| I can customise this technology to suit my needs |
| This technology’s interface is flexible |
| *Frustration* |
| The technology doesn’t let me use it in the ways I want to |
| It’s difficult to customise the technology |
| This technology’s interface is restrictive or controlling |
|  |
| **Competence** |
| *Support* |
| I find it easy to use this technology |
| I feel confident in my ability to use this technology |
| I am capable of using this technology effectively |
| *Frustration* |
| I find it hard to use this technology |
| I have doubts about my ability to use this technology |
| I struggle to use this technology effectively |
| Note. All items rated from 1 (*Strongly disagree*) to 7 (*Strongly agree*) |

**Cronbach’s alphas for each METUX subscale across the two experiments**

| Subscale | Experiment 1 | Experiment 2 (full scale) | Experiment 2 (shortened scale) |
| --- | --- | --- | --- |
| ***Life*** |  |  |  |
| Autonomy satisfaction | 0.89 | 0.89 | 0.87 |
| Autonomy frustration | 0.84 | 0.75 | 0.75 |
| Competence satisfaction | 0.93 | 0.94 | 0.9 |
| Competence frustration | 0.95 | 0.94 | 0.9 |
| Relatedness satisfaction | 0.94 | 0.93 | 0.86 |
| Relatedness frustration | 0.93 | 0.92 | 0.92 |
| ***Behaviour*** |  |  |  |
| Autonomy satisfaction | 0.87 | 0.88 | 0.89 |
| Autonomy frustration | 0.66 | 0.69 | 0.82 |
| Competence satisfaction | 0.90 | 0.92 | 0.89 |
| Competence frustration | 0.93 | 0.92 | 0.87 |
| Relatedness satisfaction | 0.93 | 0.92 | 0.92 |
| Relatedness frustration | 0.95 | 0.93 | 0.92 |
| ***Task*** |  |  |  |
| Autonomy satisfaction | 0.86 | 0.88 | 0.905 |
| Autonomy frustration | 0.69 | 0.69 | 0.84 |
| Competence satisfaction | 0.87 | 0.92 | 0.875 |
| Competence frustration | 0.90 | 0.92 | 0.885 |
| Relatedness satisfaction | 0.89 | 0.92 | 0.915 |
| Relatedness frustration | 0.96 | 0.93 | 0.935 |
| ***Interface*** |  |  |  |
| Autonomy satisfaction | 0.79 | 0.84 | 0.83 |
| Autonomy frustration | 0.86 | 0.88 | 0.85 |
| Competence satisfaction | 0.86 | 0.86 | 0.91 |
| Competence frustration | 0.92 | 0.91 | 0.91 |

# Factor loadings from Exploratory Factor analyses from Experiment 1

## Life Scale Experiment 1

| Hypothesised grouping | Item | Factor1 | Factor2 | Factor3 | Factor4 | Factor5 | Factor6 |  |
| --- | --- | --- | --- | --- | --- | --- | --- | --- |
| Autonomy  Satisfaction | choice_life | 0.01 | 0.24 | -0.01 | 0.08 | 0.03 | 0.59 |  |
|  | pursueWhatMatters_life | 0.36 | -0.17 | -0.05 | -0.01 | -0.08 | 0.57 |  |
|  | expressWhoIAm_life | -0.17 | 0.20 | -0.06 | -0.02 | 0.11 | 0.83 |  |
|  | givesMeFreedom_life | 0.01 | 0.01 | 0.02 | -0.04 | 0.02 | 0.86 |  |
|  | chooseWhatIDo_life | 0.23 | -0.06 | 0.03 | 0.02 | 0.01 | 0.71 |  |
| Autonomy  Frustration | thingsIWouldntChoose_life | 0.02 | -0.03 | 0.19 | -0.07 | 0.54 | 0.09 |  |
|  | pressuredToUse_life | 0.12 | -0.2 | 0.13 | -0.08 | 0.54 | -0.06 |  |
|  | harderToPursueWhatMatters_life | -0.06 | 0.18 | -0.13 | -0.01 | 0.92 | 0.01 |  |
|  | impairsChoice_life | -0.05 | 0.04 | -0.15 | 0.1 | 0.91 | 0.12 |  |
|  | obligedToUse_life | 0.14 | -0.11 | 0.03 | 0.05 | 0.58 | -0.15 |  |
| Competence Satisfaction | confidentInAbilities_life | 0.72 | 0.14 | 0.17 | -0.04 | -0.07 | 0.01 |  |
|  | competent_life | 0.80 | 0.10 | 0.07 | -0.02 | -0.02 | -0.03 |  |
|  | operateEffectively_life | 0.75 | -0.10 | -0.09 | 0.05 | 0.00 | 0.10 |  |
|  | completeDifficultTasks_life | 0.94 | -0.12 | -0.13 | 0.03 | 0.05 | -0.04 |  |
|  | capableAchieveGoals_life | 0.94 | -0.03 | -0.03 | 0.01 | -0.02 | -0.05 |  |
|  | learnAndGrow_life | 0.72 | 0.09 | 0.01 | 0.01 | -0.05 | -0.01 |  |
|  | improveAbilities_life | 0.89 | -0.03 | -0.02 | -0.02 | -0.01 | -0.09 |  |
| Competence Frustration | insecureAboutAbilities_life | -0.04 | 0.16 | 0.16 | 0.46 | 0.16 | -0.04 |  |
|  | doubtCapable_life | -0.02 | 0.1 | 0.17 | 0.50 | 0.17 | -0.02 |  |
|  | difficultToOvercomeChallenges_life | 0.06 | -0.06 | 0.04 | 0.83 | 0.02 | 0.00 |  |
|  | difficultToAchieveGoals_life | 0.03 | -0.06 | -0.03 | 0.95 | 0.00 | -0.02 |  |
|  | hindersLearnAndGrow_life | -0.01 | -0.02 | -0.01 | 0.98 | -0.09 | 0.00 |  |
|  | difficultToImprove_life | 0.03 | -0.06 | 0.01 | 0.97 | -0.06 | 0.00 |  |
|  | difficultToOperate_life | -0.08 | 0.06 | 0.03 | 0.73 | 0.14 | -0.02 |  |
| Relatedness Satisfaction | feelClose_life | -0.21 | 0.92 | 0.06 | 0.02 | 0.05 | 0.05 |  |
|  | feelConnected_life | -0.13 | 0.93 | 0.06 | -0.04 | 0.01 | 0.06 |  |
|  | feelOthersCare_life | -0.01 | 0.96 | 0.09 | -0.01 | 0.04 | -0.05 |  |
|  | feelPartLargerCommunity_life | 0.11 | 0.80 | 0.00 | -0.1 | 0.08 | 0.00 |  |
|  | easierToHelpOthers_life | 0.08 | 0.72 | -0.01 | 0.03 | -0.04 | 0.04 |  |
|  | feelSomethingToContribute_life | 0.26 | 0.60 | -0.05 | 0.02 | 0.00 | 0.06 |  |
|  | feelSupported_life | 0.22 | 0.73 | -0.04 | -0.01 | 0.03 | -0.06 |  |
| Relatedness Frustration | harderToConnect_life | 0.01 | -0.14 | 0.55 | 0.04 | -0.01 | 0.11 |  |
|  | feelPeopleDontCare_life | -0.01 | 0.06 | 0.87 | -0.03 | 0.00 | -0.03 |  |
|  | feelIDontMatter_life | -0.07 | 0.11 | 0.94 | -0.06 | -0.01 | -0.01 |  |
|  | feelExcluded_life | -0.07 | 0.08 | 1.03 | 0.05 | -0.19 | -0.02 |  |
|  | feelLonely_life | -0.04 | 0.12 | 0.92 | 0.02 | -0.10 | -0.03 |  |
|  | harderToHelp_life | 0.04 | -0.22 | 0.65 | 0.00 | 0.10 | 0.10 |  |
|  | harderToEngage_life | 0.11 | -0.13 | 0.73 | 0.02 | 0.01 | -0.02 |  |
| *Note*. Factor loadings greater than 0.2 are highlighted in black text. | | | | | | | | |

## Behaviour Scale Experiment 1

| Hypothesised grouping | Item | Factor1 | Factor2 | Factor3 | Factor4 | Factor5 | Factor6 | |
| --- | --- | --- | --- | --- | --- | --- | --- | --- |
| Autonomy  Satisfaction | differentOptions_behaviour | -0.07 | -0.21 | 0.06 | -0.13 | 0.73 | 0.07 | |
|  | givesMeFreedom_behaviour | -0.01 | 0.11 | -0.08 | 0.03 | 0.79 | -0.19 | |
|  | haveMoreChoice_behaviour | -0.04 | 0.12 | -0.01 | -0.07 | 0.95 | -0.13 | |
|  | MakesMeWantTo_behaviour | 0.00 | -0.10 | 0.11 | 0.27 | 0.44 | 0.15 | |
|  | alignsWithValues_behaviour | 0.03 | -0.06 | 0.10 | 0.23 | 0.36 | 0.01 | |
| Autonomy Frustration | notEnoughChoice_behaviour | -0.11 | -0.07 | 0.06 | 0.02 | -0.32 | 0.70 | |
|  | forcedWaysIDontLike_behaviour | 0.08 | 0.17 | -0.04 | -0.10 | 0.03 | 0.58 | |
|  | restrictsOptions_behaviour | -0.05 | -0.08 | -0.04 | -0.09 | -0.08 | 0.86 | |
|  | feelPressure_behaviour | 0.18 | 0.19 | -0.15 | 0.27 | 0.06 | 0.37 | |
|  | conflictsWithValues_behaviour | 0.09 | 0.37 | 0.11 | -0.02 | 0.03 | 0.27 | |
| Competence Satisfaction | easierToDo_behaviour | -0.07 | 0.06 | -0.04 | 0.79 | -0.01 | -0.06 | |
|  | confidentInAbility_behaviour | 0.00 | -0.08 | 0.04 | 0.63 | 0.11 | 0.07 | |
|  | helpsMeDoEffectively_behaviour | -0.04 | 0.06 | -0.05 | 0.96 | -0.11 | -0.08 | |
|  | usefulFor_behaviour | -0.03 | -0.04 | -0.03 | 0.89 | -0.13 | -0.06 | |
|  | helpsMeGetBetter_behaviour | 0.07 | -0.08 | 0.00 | 0.73 | 0.10 | 0.07 | |
| Competence Frustration | makesItDifficult_behaviour | -0.02 | 0.62 | 0.04 | -0.17 | -0.03 | 0.07 | |
|  | getsInTheWay_behaviour | 0.00 | 0.71 | 0.02 | -0.13 | 0.09 | 0.13 | |
|  | struggleToUse_behaviour | -0.09 | 0.86 | -0.02 | -0.03 | 0.04 | 0.00 | |
|  | loseConfidence_behaviour | 0.02 | 0.99 | -0.01 | 0.05 | 0.03 | -0.07 | |
|  | harderToImprove_behaviour | 0.05 | 0.99 | -0.01 | 0.03 | 0.00 | -0.14 | |
| Relatedness Satisfaction | feelClose_behaviour | -0.01 | 0.02 | 0.90 | -0.07 | 0.04 | 0.01 | |
|  | feelConnected_behaviour | -0.02 | -0.02 | 0.97 | -0.05 | -0.07 | -0.04 | |
|  | feelPeopleCare_behaviour | -0.01 | 0.00 | 0.93 | -0.07 | 0.03 | 0.03 | |
|  | feelPartOfCommunity_behaviour | 0.07 | -0.10 | 0.77 | 0.03 | 0.02 | 0.06 | |
|  | easierToHelpOthers_behaviour | 0.02 | 0.16 | 0.69 | 0.23 | -0.07 | -0.13 | |
| Relatedness Frustration | feelDisconnected_behaviour | 0.80 | -0.03 | -0.07 | -0.06 | 0.02 | -0.02 | |
|  | feelOthersDontCare_behaviour | 0.83 | 0.11 | 0.08 | -0.04 | -0.08 | -0.08 | |
|  | feelExcluded_behaviour | 0.96 | -0.05 | 0.00 | 0.01 | -0.05 | -0.03 | |
|  | feelLonely_behaviour | 0.98 | -0.16 | -0.01 | -0.04 | 0.01 | 0.02 | |
|  | feelIDontMatter_behaviour | 0.85 | 0.14 | 0.03 | 0.05 | -0.01 | -0.05 | |
|  | harderToHelpOthers_behaviour | 0.73 | 0.05 | -0.06 | -0.04 | 0.03 | 0.02 | |
| *Note*. Factor loadings greater than 0.2 are highlighted in black text. | | | | | | | |  |

# Task Scale (Sending messages on Facebook)

| Hypothesised grouping | Item | Factor1 | Factor2 | Factor3 | Factor4 | Factor5 | Factor6 | |
| --- | --- | --- | --- | --- | --- | --- | --- | --- |
| Autonomy  Satisfaction | differentOptions_task1 | -0.21 | -0.09 | 0.86 | 0.06 | 0.00 | -0.02 | |
|  | givesMeFreedom_task1 | 0.05 | -0.05 | 0.63 | -0.41 | 0.08 | 0.14 | |
|  | haveMoreChoice_task1 | -0.05 | -0.09 | 0.97 | -0.1 | 0.19 | -0.07 | |
|  | makesMeWantTo_task1 | 0.48 | -0.03 | 0.52 | 0.22 | 0.08 | 0.04 | |
|  | alignsWithValues_task1 | 0.37 | 0.01 | 0.31 | -0.18 | 0.06 | -0.03 | |
| Autonomy Frustration | notEnoughChoice_task1 | 0.01 | -0.07 | -0.43 | 0.52 | 0.09 | -0.15 | |
|  | forcedToDo_task1 | 0.12 | 0.06 | -0.12 | 0.67 | 0.27 | -0.03 | |
|  | restrictsOptions_task1 | 0.13 | -0.07 | -0.34 | 0.8 | 0.00 | -0.07 | |
|  | feelPressure_task1 | -0.03 | 0.14 | 0.18 | 0.54 | -0.05 | 0.09 | |
|  | conflictsWithValues_task1 | -0.26 | 0.07 | 0.22 | 0.54 | -0.06 | 0.05 | |
| Competence Satisfaction | easierToDo_task1 | 0.26 | 0.09 | 0.00 | -0.07 | -0.50 | 0.09 | |
|  | confidentInAbility_task1 | 0.44 | 0.24 | -0.07 | -0.09 | -0.33 | -0.24 | |
|  | helpsMeDoEffectively_task1 | 0.25 | 0.09 | 0.08 | -0.11 | -0.41 | -0.14 | |
|  | usefulFor_task1 | 0.26 | 0.02 | 0.12 | -0.16 | -0.32 | -0.03 | |
|  | helpsMeGetBetter_task1 | 0.35 | 0.07 | 0.28 | 0.14 | -0.22 | -0.10 | |
| Competence Frustration | makesItDifficult_task1 | -0.04 | -0.03 | 0.10 | 0.29 | 0.45 | 0.19 | |
|  | getsInTheWay_task1 | 0.18 | 0.34 | 0.18 | 0.07 | 0.74 | 0.11 | |
|  | struggleToUse_task1 | 0.04 | 0.25 | -0.07 | 0.12 | 0.05 | 0.51 | |
|  | loseConfidence_task1 | -0.04 | 0.00 | 0.05 | -0.08 | 0.04 | 1.02 | |
|  | harderToImprove_task1 | 0.15 | 0.14 | -0.22 | -0.03 | 0.13 | 0.64 | |
| Relatedness Satisfaction | feelClose_task1 | 1.06 | -0.05 | -0.13 | 0.05 | 0.09 | -0.02 | |
|  | feelConnected_task1 | 1.01 | -0.16 | -0.14 | 0.00 | 0.14 | 0.03 | |
|  | feelPeopleCare_task1 | 0.84 | -0.02 | 0.01 | 0.10 | 0.02 | 0.03 | |
|  | feelPartOfCommunity_task1 | 0.71 | -0.02 | 0.04 | 0.12 | -0.01 | 0.04 | |
|  | easierToHelpOthers_task1 | 0.48 | -0.05 | -0.01 | -0.3 | -0.07 | 0.16 | |
| Relatedness Frustration | feelDisconnected_task1 | -0.11 | 0.71 | 0.13 | -0.12 | 0.50 | -0.05 | |
|  | feelPeopleDontCare_task1 | 0.10 | 0.97 | 0.05 | -0.12 | 0.55 | -0.23 | |
|  | feelExcluded_task1 | -0.26 | 0.80 | -0.05 | -0.05 | -0.13 | 0.15 | |
|  | feelLonely_task1 | -0.06 | 0.85 | -0.11 | 0.00 | -0.08 | 0.15 | |
|  | feelIDontMatter_task1 | 0.04 | 0.90 | -0.19 | 0.12 | 0.04 | -0.08 | |
|  | harderToHelpOthers_task1 | -0.04 | 0.58 | -0.01 | 0.24 | 0.07 | 0.04 | |
| *Note*. Factor loadings greater than 0.2 are highlighted in black text. | | | | | | | |  |

# Task Scale (Sharing photos on Facebook)

| Hypothesised grouping | Item | Factor1 | Factor2 | Factor3 | Factor4 | Factor5 | Factor6 | |
| --- | --- | --- | --- | --- | --- | --- | --- | --- |
| Autonomy  Satisfaction | differentOptions_task1 | 0.14 | -0.12 | -0.01 | 0.99 | -0.12 | -0.11 | |
|  | givesMeFreedom_task1 | -0.12 | 0.07 | -0.06 | 0.75 | 0.08 | -0.19 | |
|  | haveMoreChoice_task1 | -0.04 | -0.09 | -0.08 | 0.92 | -0.02 | -0.09 | |
|  | makesMeWantTo_task1 | 0.02 | -0.07 | 0.29 | 0.07 | 0.48 | 0.08 | |
|  | alignsWithValues_task1 | 0.15 | -0.06 | -0.01 | 0.04 | 0.80 | -0.07 | |
| Autonomy Frustration | notEnoughChoice_task1 | 0.06 | -0.12 | -0.03 | -0.42 | -0.07 | 0.55 | |
|  | forcedToDo_task1 | -0.04 | 0.22 | -0.13 | -0.1 | -0.06 | 0.65 | |
|  | restrictsOptions_task1 | 0.22 | -0.18 | -0.09 | -0.18 | -0.07 | 0.65 | |
|  | feelPressure_task1 | -0.11 | 0.15 | 0.12 | 0.08 | -0.47 | 0.28 | |
|  | conflictsWithValues_task1 | 0.07 | 0.21 | 0.10 | 0.29 | -0.65 | 0.26 | |
| Competence Satisfaction | easierToDo_task1 | -0.68 | 0.07 | 0.02 | 0.26 | -0.06 | 0.13 | |
|  | confidentInAbility_task1 | -0.33 | 0.11 | -0.07 | 0.18 | 0.51 | 0.16 | |
|  | helpsMeDoEffectively_task1 | -0.57 | 0.13 | 0.09 | 0.00 | 0.44 | 0.06 | |
|  | usefulFor_task1 | -0.53 | -0.01 | 0.41 | -0.05 | 0.14 | 0.12 | |
|  | helpsMeGetBetter_task1 | 0.03 | -0.02 | 0.31 | 0.29 | 0.31 | 0.04 | |
| Competence Frustration | makesItDifficult_task1 | 0.83 | -0.12 | 0.04 | 0.11 | -0.21 | 0.11 | |
|  | getsInTheWay_task1 | 0.98 | 0.03 | -0.07 | 0.06 | 0.19 | 0.10 | |
|  | struggleToUse_task1 | 0.84 | 0.02 | 0.07 | -0.04 | 0.02 | 0.03 | |
|  | loseConfidence_task1 | 0.78 | 0.32 | 0.03 | 0.04 | 0.08 | -0.04 | |
|  | harderToImprove_task1 | 0.59 | 0.17 | -0.04 | -0.01 | 0.12 | 0.27 | |
| Relatedness Satisfaction | feelClose_task1 | 0.01 | 0.02 | 1.11 | -0.08 | -0.17 | -0.18 | |
|  | feelConnected_task1 | -0.07 | -0.01 | 1.06 | -0.08 | -0.18 | -0.11 | |
|  | feelPeopleCare_task1 | 0.01 | 0.08 | 0.78 | -0.01 | 0.09 | -0.06 | |
|  | feelPartOfCommunity_task1 | 0.01 | -0.13 | 0.69 | -0.02 | 0.12 | 0.17 | |
|  | easierToHelpOthers_task1 | 0.38 | 0.02 | 0.32 | 0.08 | 0.38 | 0.02 | |
| Relatedness Frustration | feelDisconnected_task1 | 0.07 | 0.82 | -0.12 | -0.17 | 0.08 | -0.09 | |
|  | feelPeopleDontCare_task1 | 0.02 | 0.85 | -0.03 | 0.04 | 0.08 | -0.03 | |
|  | feelExcluded_task1 | -0.05 | 0.96 | -0.04 | -0.06 | -0.03 | -0.03 | |
|  | feelLonely_task1 | 0.00 | 0.97 | 0.10 | -0.08 | -0.12 | -0.15 | |
|  | feelIDontMatter_task1 | -0.03 | 0.83 | -0.02 | 0.05 | -0.05 | 0.13 | |
|  | harderToHelpOthers_task1 | 0.18 | 0.57 | 0.07 | 0.05 | -0.14 | 0.21 | |
| *Note*. Factor loadings greater than 0.2 are highlighted in black text. | | | | | | | |  |

# Task Scale (Submitting assignments on virtual learning systems)

| Hypothesised grouping | Item | Factor1 | Factor2 | Factor3 | Factor4 | Factor5 | Factor6 | |
| --- | --- | --- | --- | --- | --- | --- | --- | --- |
| Autonomy  Satisfaction | differentOptions_task1 | -0.08 | 0.00 | -0.11 | -0.05 | 0.87 | -0.01 | |
|  | givesMeFreedom_task1 | -0.01 | -0.05 | 0.01 | 0.11 | 0.82 | -0.23 | |
|  | haveMoreChoice_task1 | -0.01 | -0.07 | -0.1 | 0.00 | 0.99 | -0.13 | |
|  | makesMeWantTo_task1 | 0.02 | 0.15 | 0.25 | -0.12 | 0.28 | 0.14 | |
|  | alignsWithValues_task1 | -0.08 | 0.06 | 0.10 | 0.01 | 0.47 | 0.12 | |
| Autonomy Frustration | notEnoughChoice_task1 | -0.09 | -0.08 | -0.03 | -0.03 | -0.16 | 0.59 | |
|  | forcedToDo_task1 | 0.07 | -0.03 | -0.22 | -0.05 | -0.01 | 0.70 | |
|  | restrictsOptions_task1 | -0.01 | 0.01 | 0.02 | 0.02 | -0.34 | 0.68 | |
|  | feelPressure_task1 | 0.03 | -0.12 | 0.15 | 0.09 | 0.09 | 0.68 | |
|  | conflictsWithValues_task1 | 0.19 | 0.04 | -0.02 | 0.42 | 0.10 | 0.22 | |
| Competence Satisfaction | easierToDo_task1 | 0.06 | -0.02 | 0.74 | -0.15 | -0.03 | -0.11 | |
|  | confidentInAbility_task1 | 0.07 | 0.03 | 0.88 | 0.13 | -0.06 | -0.10 | |
|  | helpsMeDoEffectively_task1 | 0.02 | 0.01 | 0.86 | -0.12 | -0.10 | 0.03 | |
|  | usefulFor_task1 | -0.12 | -0.06 | 0.65 | -0.23 | -0.08 | 0.04 | |
|  | helpsMeGetBetter_task1 | -0.06 | 0.08 | 0.64 | 0.14 | 0.14 | 0.18 | |
| Competence Frustration | makesItDifficult_task1 | -0.11 | 0.10 | -0.48 | 0.29 | 0.04 | 0.22 | |
|  | getsInTheWay_task1 | -0.03 | 0.14 | -0.17 | 0.50 | -0.10 | 0.14 | |
|  | struggleToUse_task1 | -0.09 | -0.06 | 0.04 | 0.96 | -0.03 | -0.02 | |
|  | loseConfidence_task1 | 0.00 | -0.03 | 0.00 | 0.89 | 0.02 | 0.00 | |
|  | harderToImprove_task1 | 0.12 | -0.01 | 0.02 | 0.84 | 0.05 | -0.06 | |
| Relatedness Satisfaction | feelClose_task1 | 0.03 | 0.85 | -0.07 | 0.12 | -0.02 | -0.17 | |
|  | feelConnected_task1 | -0.02 | 0.98 | -0.01 | 0.00 | -0.12 | -0.04 | |
|  | feelPeopleCare_task1 | 0.11 | 0.82 | -0.02 | -0.05 | 0.04 | -0.05 | |
|  | feelPartOfCommunity_task1 | -0.17 | 0.80 | 0.10 | -0.10 | -0.03 | 0.18 | |
|  | easierToHelpOthers_task1 | 0.04 | 0.72 | 0.01 | -0.06 | 0.07 | -0.04 | |
| Relatedness Frustration | feelDisconnected_task1 | 0.73 | 0.09 | -0.02 | -0.08 | -0.02 | 0.10 | |
|  | feelPeopleDontCare_task1 | 0.85 | 0.03 | 0.07 | 0.15 | -0.09 | -0.10 | |
|  | feelExcluded_task1 | 0.94 | 0.01 | 0.04 | 0.02 | -0.01 | -0.03 | |
|  | feelLonely_task1 | 0.9 | 0.00 | -0.04 | -0.12 | -0.03 | 0.08 | |
|  | feelIDontMatter_task1 | 0.93 | -0.08 | 0.07 | 0.08 | -0.05 | -0.07 | |
|  | harderToHelpOthers_task1 | 0.65 | -0.04 | -0.09 | -0.18 | 0.21 | 0.20 | |
| *Note*. Factor loadings greater than 0.2 are highlighted in black text. | | | | | | | |  |

# Task Scale (Viewing course materials on virtual learning systems)

| Hypothesised grouping | Item | Factor1 | Factor2 | Factor3 | Factor4 | Factor5 | Factor6 | |
| --- | --- | --- | --- | --- | --- | --- | --- | --- |
| Autonomy  Satisfaction | differentOptions_task1 | -0.04 | -0.11 | -0.02 | -0.16 | 0.07 | 0.80 | |
|  | givesMeFreedom_task1 | 0.00 | -0.02 | -0.02 | 0.14 | -0.22 | 0.80 | |
|  | haveMoreChoice_task1 | -0.02 | -0.16 | -0.02 | -0.01 | -0.09 | 0.99 | |
|  | makesMeWantTo_task1 | 0.03 | 0.33 | 0.03 | -0.01 | 0.13 | 0.44 | |
|  | alignsWithValues_task1 | 0.03 | 0.29 | 0.15 | 0.02 | 0.11 | 0.38 | |
| Autonomy Frustration | notEnoughChoice_task1 | -0.04 | -0.03 | -0.08 | 0.08 | 0.73 | -0.03 | |
|  | forcedToDo_task1 | -0.04 | -0.10 | -0.11 | -0.01 | 0.81 | -0.03 | |
|  | restrictsOptions_task1 | -0.07 | -0.02 | -0.01 | -0.09 | 0.87 | -0.19 | |
|  | feelPressure_task1 | 0.12 | 0.05 | 0.06 | -0.01 | 0.70 | 0.12 | |
|  | conflictsWithValues_task1 | 0.16 | 0.03 | 0.14 | 0.24 | 0.45 | -0.06 | |
| Competence Satisfaction | easierToDo_task1 | -0.01 | 0.80 | 0.03 | -0.03 | -0.11 | -0.09 | |
|  | confidentInAbility_task1 | 0.03 | 0.86 | 0.06 | -0.08 | 0.03 | -0.09 | |
|  | helpsMeDoEffectively_task1 | -0.01 | 0.87 | -0.06 | -0.17 | -0.03 | -0.11 | |
|  | usefulFor_task1 | -0.04 | 0.86 | -0.10 | -0.08 | -0.01 | -0.03 | |
|  | helpsMeGetBetter_task1 | -0.02 | 0.82 | -0.01 | 0.17 | -0.08 | 0.03 | |
| Competence Frustration | makesItDifficult_task1 | -0.12 | -0.08 | -0.05 | 0.55 | 0.24 | -0.01 | |
|  | getsInTheWay_task1 | -0.06 | -0.11 | 0.13 | 0.64 | 0.22 | -0.05 | |
|  | struggleToUse_task1 | -0.08 | -0.02 | -0.10 | 0.89 | -0.03 | 0.07 | |
|  | loseConfidence_task1 | 0.04 | -0.07 | 0.01 | 0.79 | -0.01 | -0.04 | |
|  | harderToImprove_task1 | 0.09 | -0.14 | -0.05 | 0.77 | -0.06 | -0.02 | |
| Relatedness Satisfaction | feelClose_task1 | 0.08 | 0.05 | 0.79 | 0.09 | 0.03 | -0.04 | |
|  | feelConnected_task1 | -0.02 | 0.00 | 0.91 | 0.02 | -0.06 | 0.00 | |
|  | feelPeopleCare_task1 | 0.05 | -0.06 | 0.84 | -0.04 | -0.02 | 0.05 | |
|  | feelPartOfCommunity_task1 | -0.05 | -0.03 | 0.89 | -0.06 | -0.03 | -0.03 | |
|  | easierToHelpOthers_task1 | -0.12 | -0.07 | 0.83 | -0.09 | -0.03 | -0.03 | |
| Relatedness Frustration | feelDisconnected_task1 | 0.83 | 0.00 | -0.10 | -0.17 | 0.13 | -0.01 | |
|  | feelPeopleDontCare_task1 | 0.89 | 0.04 | 0.08 | 0.06 | -0.03 | -0.04 | |
|  | feelExcluded_task1 | 0.97 | -0.03 | -0.05 | -0.06 | -0.02 | 0.03 | |
|  | feelLonely_task1 | 0.95 | -0.01 | -0.03 | -0.06 | -0.03 | 0.00 | |
|  | feelIDontMatter_task1 | 0.88 | 0.02 | 0.03 | 0.09 | -0.05 | -0.04 | |
|  | harderToHelpOthers_task1 | 0.72 | -0.10 | 0.01 | 0.13 | -0.09 | 0.01 | |
| *Note*. Factor loadings greater than 0.2 are highlighted in black text. | | | | | | | |  |

| Hypothesised grouping | Item | Factor1 | Factor2 | Factor3 | Factor4 |
| --- | --- | --- | --- | --- | --- |
| Autonomy  Satisfaction | usefulOptions_interface | 0.04 | 0.4 | 0.08 | 0.51 |
|  | givesMeFreedom_interface | 0.06 | 0.48 | -0.13 | 0.33 |
|  | canCustomise_interface | 0.01 | 0.93 | 0.13 | 0.03 |
|  | interfaceFlexible_interface | 0.09 | 0.93 | 0.13 | 0.15 |
| Autonomy Frustration | doesntProvideOptions_interface | -0.08 | 0.04 | 0.91 | 0.05 |
|  | wontLetMeUse_interface | -0.03 | -0.06 | 0.82 | -0.01 |
|  | cantCustomise_interface | 0.06 | -0.73 | 0.14 | 0.13 |
|  | restrictiveOrControlling_interface | 0.08 | -0.56 | 0.27 | 0.05 |
| Competence Satisfaction | easyToUse_interface | -0.54 | -0.02 | -0.05 | 0.44 |
|  | confidentInAbility_interface | -0.64 | -0.19 | 0.07 | 0.54 |
|  | capableOfUsing_interface | -0.67 | -0.14 | 0 | 0.45 |
|  | interfaceIntuitive_interface | -0.14 | 0.07 | -0.26 | 0.27 |
| Competence Frustration | hardToUse_interface | 0.82 | -0.06 | -0.01 | -0.07 |
|  | doubtsAboutAbility_interface | 0.96 | -0.01 | -0.06 | 0.07 |
|  | struggleToUse_interface | 0.9 | -0.06 | -0.02 | 0.05 |
|  | interfaceConfusing_interface | 0.59 | -0.21 | 0.07 | -0.05 |

**Correlations from Experiment 2 between technology evaluations and need satisfaction and frustration ratings for each sphere**

| Subscale | Star rating | Satisfaction with technology | Willingness to recommend to others |
| --- | --- | --- | --- |
| ***Life*** |  |  |  |
| Autonomy satisfaction | 0.40 | 0.42 | 0.46 |
| Autonomy frustration | -0.19 | -0.19 | -0.14 |
| Competence satisfaction | 0.36 | 0.39 | 0.38 |
| Competence frustration | -0.25 | -0.26 | -0.22 |
| Relatedness satisfaction | 0.30 | 0.32 | 0.37 |
| Relatedness frustration | -0.23 | -0.23 | -0.23 |
| ***Behavior*** |  |  |  |
| Autonomy satisfaction | 0.36 | 0.33 | 0.40 |
| Autonomy frustration | -0.36 | -0.34 | -0.35 |
| Competence satisfaction | 0.35 | 0.38 | 0.37 |
| Competence frustration | -0.27 | -0.30 | -0.31 |
| Relatedness satisfaction | 0.24 | 0.23 | 0.29 |
| Relatedness frustration | -0.20 | -0.22 | -0.23 |
| ***Task*** |  |  |  |
| Autonomy satisfaction | 0.33 | 0.31 | 0.37 |
| Autonomy frustration | -0.38 | -0.37 | -0.37 |
| Competence satisfaction | 0.45 | 0.47 | 0.48 |
| Competence frustration | -0.35 | -0.35 | -0.33 |
| Relatedness satisfaction | 0.31 | 0.28 | 0.37 |
| Relatedness frustration | -0.22 | -0.25 | -0.26 |
| ***Interface*** |  |  |  |
| Autonomy satisfaction | 0.45 | 0.44 | 0.46 |
| Autonomy frustration | -0.38 | -0.38 | -0.37 |
| Competence satisfaction | 0.39 | 0.41 | 0.38 |
| Competence frustration | -0.32 | -0.32 | -0.30 |
| *Note*. All correlations are significantly different from zero. | | | |

# Correlations between scale-specific DVs and psychological needs

## Life scale

| Dependent Measure | Autonomy satisfaction | Autonomy frustration | Competence satisfaction | Competence frustration | Relatedness satisfaction | Relatedness frustration |
| --- | --- | --- | --- | --- | --- | --- |
| This technology makes my life better | 0.48 | -0.15 | 0.53 | -0.21 | 0.31 | -0.20 |
| [technology] helps me enjoy life | 0.55 | -0.12 | 0.40 | -0.17 | 0.45 | -0.19 |
| [technology] is useful to me | 0.39 | -0.16 | 0.49 | -0.22 | 0.29 | -0.19 |
| How often do you use [technology]? | 0.21 | -0.01 | 0.07 | -0.05 | 0.20 | -0.09 |
| Approximately how many hours per week do you spend using [technology]? | 0.20 | 0.04 | 0.15 | -0.02 | 0.18 | -0.03 |

## Behaviour Scale

| Dependent Measure | Autonomy satisfaction | Autonomy frustration | Competence satisfaction | Competence frustration | Relatedness satisfaction | Relatedness frustration |
| --- | --- | --- | --- | --- | --- | --- |
| How often do you [behaviour]? | 0.10 | -0.08 | 0.10 | -0.08 | 0.14 | -0.06 |
| In general, how easy is it for you to [behaviour]? | 0.30 | -0.34 | 0.23 | -0.28 | 0.40 | -0.24 |
| In general, how much choice do you feel you have over how you [behaviour]? | 0.41 | -0.40 | 0.19 | -0.26 | 0.37 | -0.25 |
| I enjoy [behaviour] | 0.30 | -0.24 | 0.30 | -0.24 | 0.38 | -0.21 |
| When I [behaviour], I consider it time well spent | -0.03 | 0.01 | 0.00 | 0.01 | -0.05 | 0.02 |
| [technology] helps me enjoy [behaviour] | 0.53 | -0.37 | 0.56 | -0.34 | 0.47 | -0.23 |

## Task Scale

| Dependent Measure | Autonomy satisfaction | Autonomy frustration | Competence satisfaction | Competence frustration | Relatedness satisfaction | Relatedness frustration |
| --- | --- | --- | --- | --- | --- | --- |
| How often do you use [technology] to [task]? | 0.12 | -0.10 | 0.19 | -0.16 | 0.20 | -0.14 |
| I enjoy [task] | 0.37 | -0.35 | 0.42 | -0.35 | 0.39 | -0.26 |
| When I use [technology] to [task], I consider it time well spent | 0.44 | -0.40 | 0.50 | -0.35 | 0.41 | -0.27 |
| [technology] helps me enjoy [task] | 0.16 | -0.20 | 0.37 | -0.20 | 0.04 | -0.16 |
| *Note.* The table displays the average correlation across the two tasks | | | | | | |

**Interface Scale**

| Dependent Measure | Autonomy satisfaction | Autonomy frustration | Competence satisfaction | Competence frustration |
| --- | --- | --- | --- | --- |
| Usability score | 0.51 | -0.53 | 0.76 | -0.73 |

# Confirmatory Factor Analyses from Experiment 2

## Life scale (full)

##
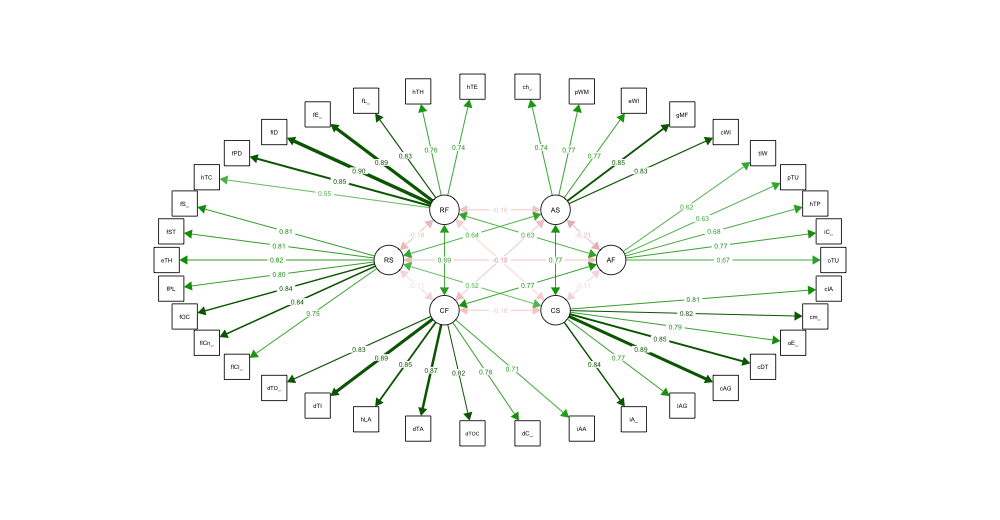


## Life scale (shortened)


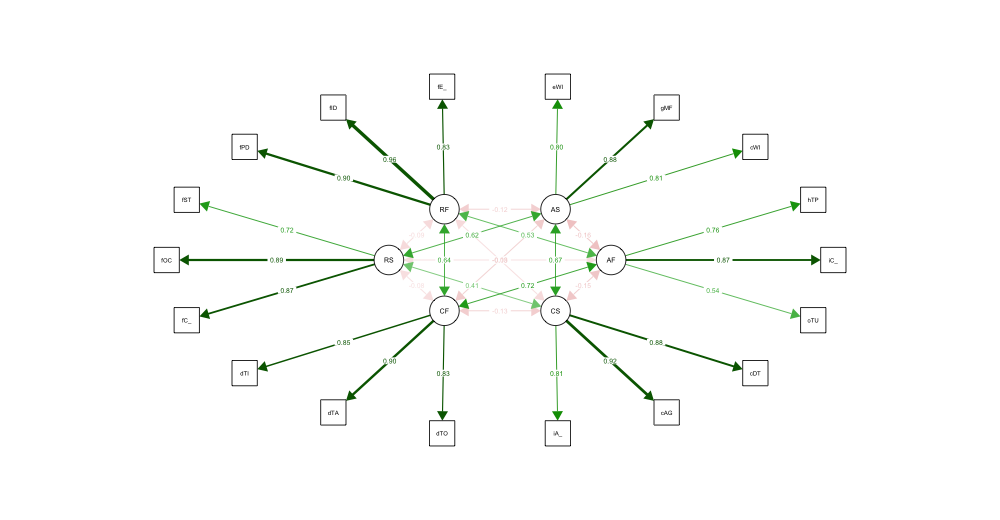


## Behaviour scale (full)


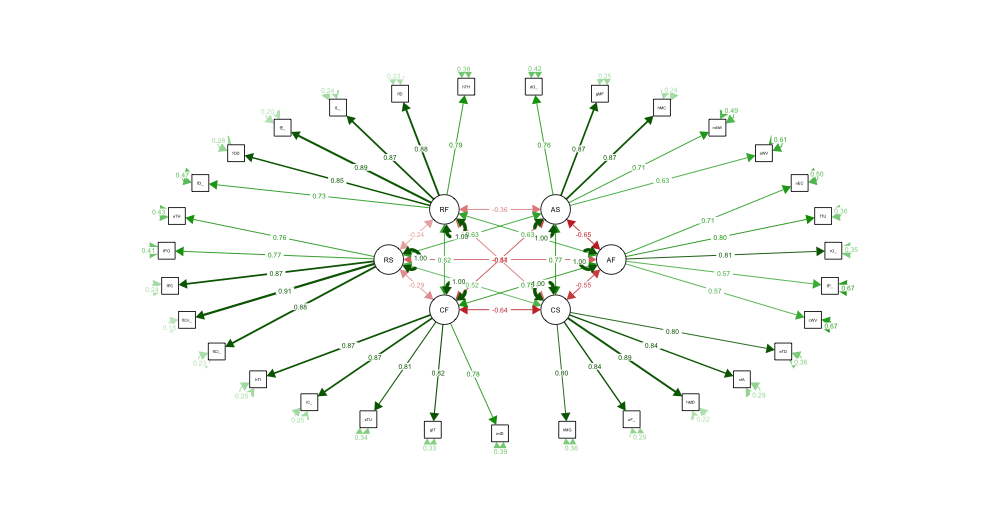


## Behaviour scale (shortened)


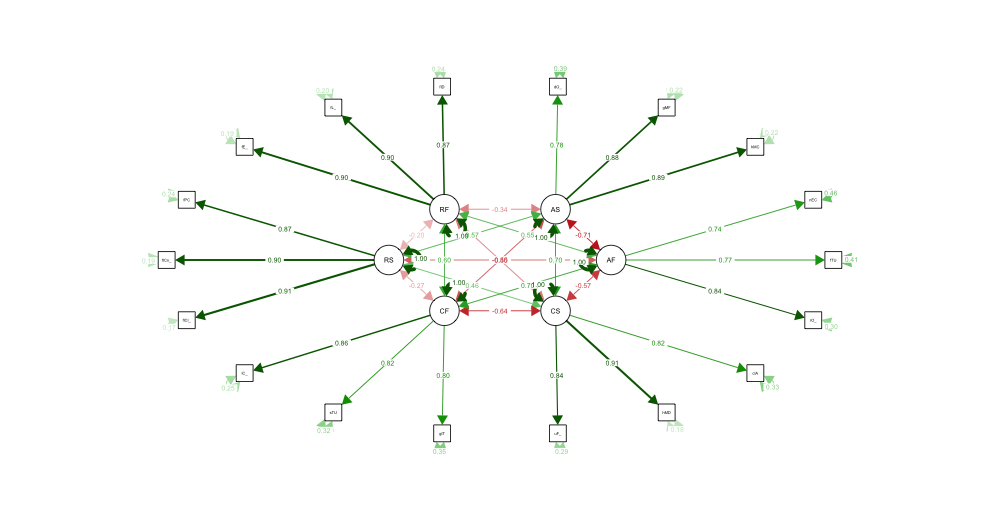


## Task scale (first task, full)

**
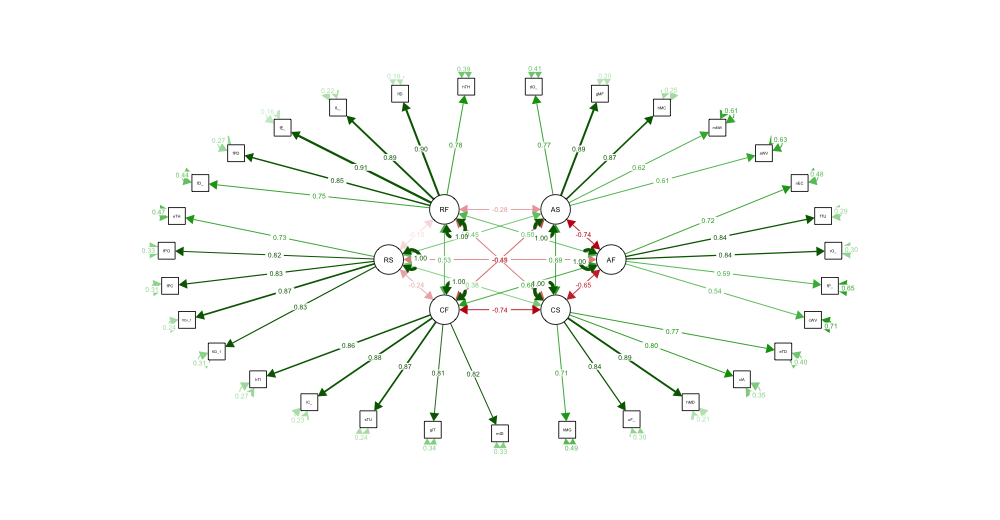
**

## Task scale (first task, shortened)

**
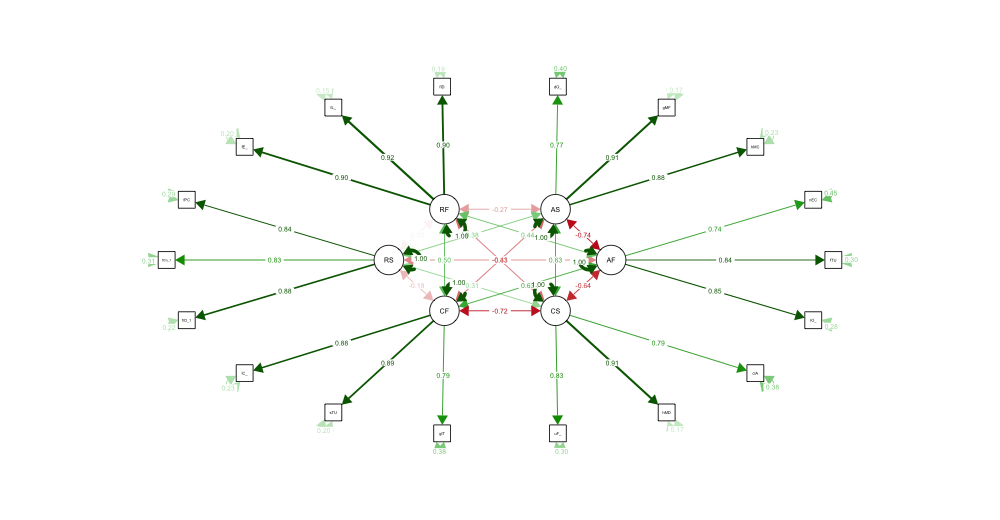
**

## Task scale (second task, full)


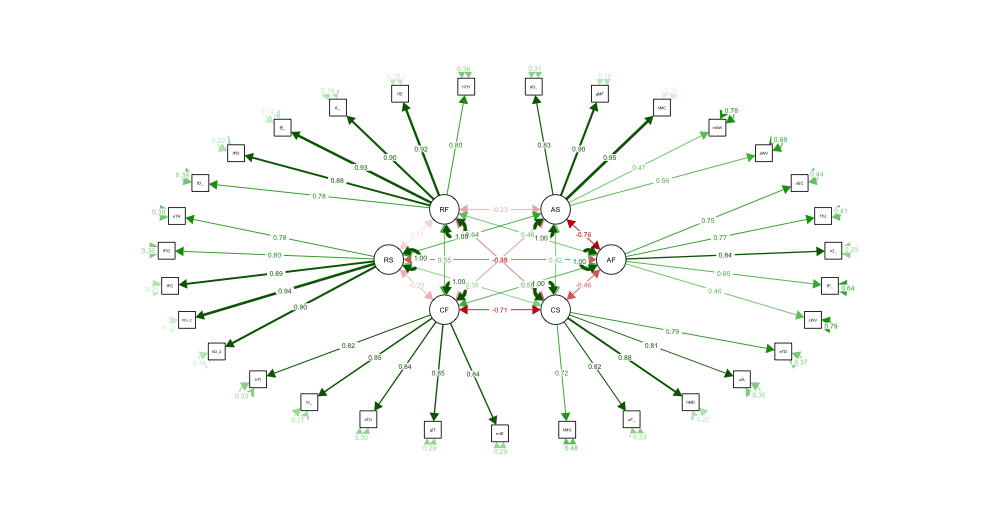


## Task scale (second task, shortened)


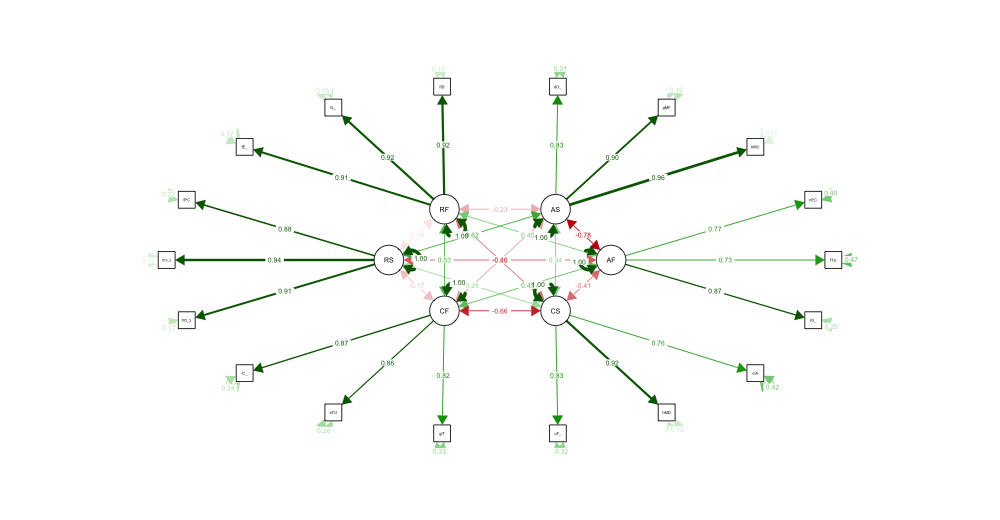


## Interface scale (full)


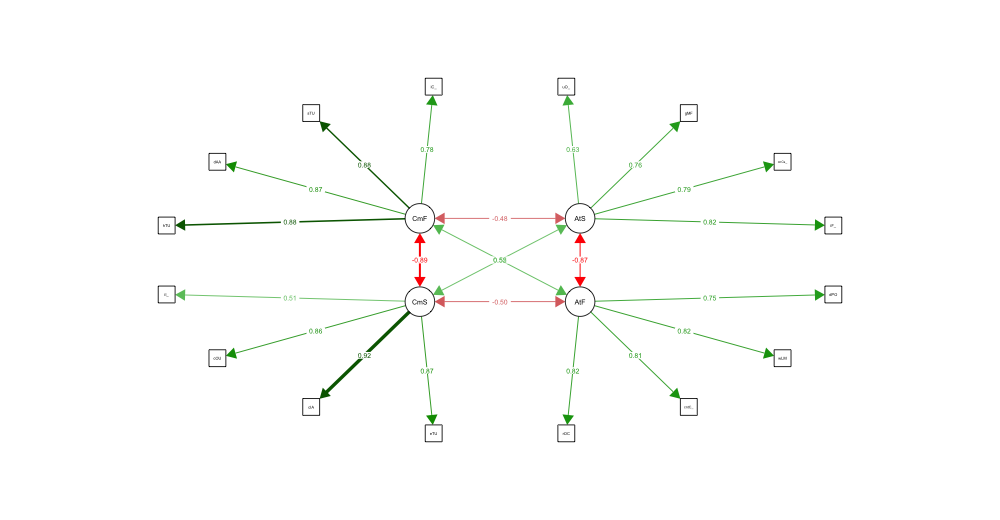


## Interface scale (shortened)


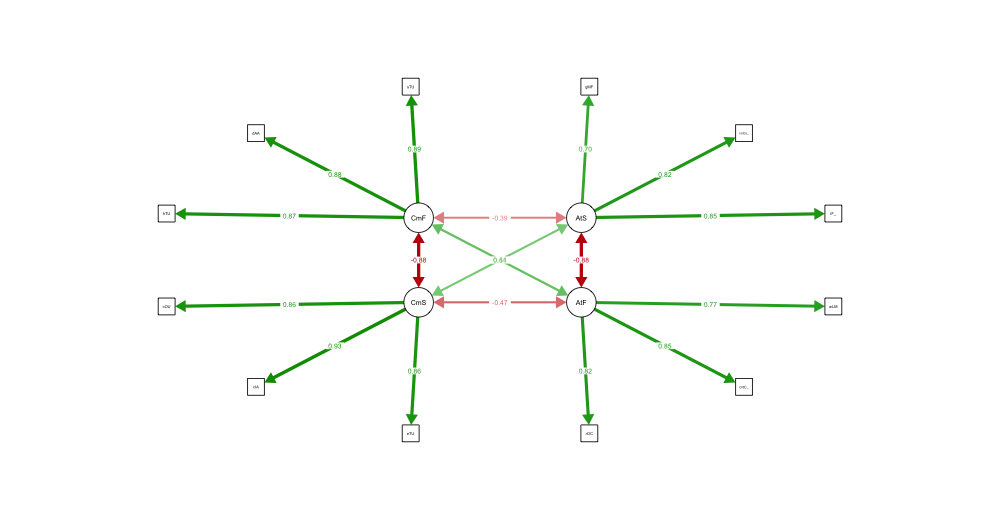

Supplement: Supplementary file 1 [file Data_Sheet_1.DOCX]
